# Electroceutically induced subthalamic high-frequency oscillations and evoked compound activity may explain the mechanism of therapeutic stimulation in Parkinson’s disease

**DOI:** 10.1038/s42003-021-01915-7

**Published:** 2021-03-23

**Authors:** Musa Ozturk, Ashwin Viswanathan, Sameer A. Sheth, Nuri F. Ince

**Affiliations:** 1 Department of Biomedical Engineering, University of Houston, Houston, TX USA; 2 Department of Neurosurgery, Baylor College of Medicine, Houston, TX USA

**Keywords:** Parkinson's disease, Parkinson's disease

## Abstract

Despite having remarkable utility in treating movement disorders, the lack of understanding of the underlying mechanisms of high-frequency deep brain stimulation (DBS) is a main challenge in choosing personalized stimulation parameters. Here we investigate the modulations in local field potentials induced by electrical stimulation of the subthalamic nucleus (STN) at therapeutic and non-therapeutic frequencies in Parkinson’s disease patients undergoing DBS surgery. We find that therapeutic high-frequency stimulation (130–180 Hz) induces high-frequency oscillations (~300 Hz, HFO) similar to those observed with pharmacological treatment. Along with HFOs, we also observed evoked compound activity (ECA) after each stimulation pulse. While ECA was observed in both therapeutic and non-therapeutic (20 Hz) stimulation, the HFOs were induced only with therapeutic frequencies, and the associated ECA were significantly more resonant. The relative degree of enhancement in the HFO power was related to the interaction of stimulation pulse with the phase of ECA. We propose that high-frequency STN-DBS tunes the neural oscillations to their healthy/treated state, similar to pharmacological treatment, and the stimulation frequency to maximize these oscillations can be inferred from the phase of ECA waveforms of individual subjects. The induced HFOs can, therefore, be utilized as a marker of successful re-calibration of the dysfunctional circuit generating PD symptoms.

## Abstract (web summary)

Ozturk et al investigate the modulations in local field potentials induced by electrical stimulation of the subthalamic nucleus (STN) at therapeutic and nontherapeutic frequencies in Parkinson’s disease (PD) patients undergoing DBS surgery. They find evidence to suggest that high-frequency STN-DBS tunes the neural oscillations to their healthy/treated state, similar to pharmacological treatment and thus could serve to recalibrate the dysfunctional circuitry generating PD symptoms.

## Introduction

Chronic high-frequency (>100 Hz) deep brain stimulation (DBS) is an established medical treatment for movement disorders such as Parkinson’s disease (PD) and is being explored for the treatment of many other neurological and psychiatric indications^1–3^. Yet, despite decades of clinical use, its underlying therapeutic mechanism is still unclear^1,2^. In particular, there is limited knowledge regarding the neural oscillatory modulations induced with therapeutic high-frequency stimulation (HFS). If robust and target-specific neural signatures associated with HFS can be discovered, they can both assist to uncover the mechanism of DBS therapy and open the path for the construction of adaptive therapies that can tune the stimulation parameters for individual PD patients.

The studies seeking an electrophysiological basis for the mechanisms of DBS have focused on the investigation of neuronal spiking and oscillatory activity from the basal ganglia. Early hypotheses suggested that high-frequency DBS mimics lesioning by inhibiting neuronal firing from the stimulated structure^4–8^. Others proposed that DBS therapy overrides the pathological burst-type firing with its stimulus-induced regular (tonic) pattern and thereby ameliorated parkinsonian symptoms^9–11^. This effect is not only in the stimulated structure but also travels downstream to the basal ganglia-thalamo-cortical circuit^10,12^ and creates an “informational lesion” preventing the relay of pathological firing and oscillations^13^. However, other studies suggest that DBS, by regularizing basal ganglia spiking activity, enhances the information processing and restores responsiveness of the thalamocortical cells to the incoming sensorimotor information^14–16^, indicating that rather than causing “lesioning”, DBS might exert its therapeutic effect through promotion of neural activity similar to the “healthy” state^17,18^.

Local field potentials (LFP) of the basal ganglia have long attracted interest due to their utility as a feedback modality for closed-loop DBS. Particularly in the subthalamic nucleus (STN), one of the frequently targeted structures in PD patients^19,20^, excessive beta (12–30 Hz) band oscillations are considered as the hallmark^21,22^ and have shown to diminish with DBS^12,23,24^ and dopaminergic medication^23,25,26^. More recently, the broadband high-frequency oscillations (200–450 Hz, HFO) of LFPs and cross-frequency coupling between beta and HFO bands^27–30^ have been identified as important markers in PD electrophysiology. Although the pharmaceutical modulations of the LFP bands (e.g., suppression of beta and enhancement in the HFO bands) have been well-documented^29–33^, the large stimulus artifact observed during DBS have hindered further investigation of these biomarkers, especially in the HFO range, for closed-loop neuromodulation applications^34^. Consequently, the contribution of LFPs in uncovering the mechanisms of DBS have been limited due to the inability to record these oscillations during stimulation.

With these motivations, we established an intraoperative system to record LFPs during acute stimulation of STN in PD patients undergoing DBS surgery. We hypothesized that HFS exerts its therapeutic effect by modulating oscillatory neural activity in the STN, similar to the effect of pharmaceutical treatment. To test this hypothesis, we recorded LFPs from microelectrodes intraoperatively and studied their modulation during multiple low- and HFS paradigms, both outside and within the STN. We observed that high-frequency therapeutic DBS (>100 Hz) induced HFO activity similar to the reports in the pharmacologically treated patients and healthy animals. In conjunction, we noted an evoked activity after each stimulus pulse, which was more resonant with the HFS. More interestingly, the strength of induced HFO was related to the interaction of stimulation pulses with the phase of the evoked waveform, indicating that both measures and their characteristics can be used functionally to optimize electroceutical therapy.

## Results

**Fig. 1:**
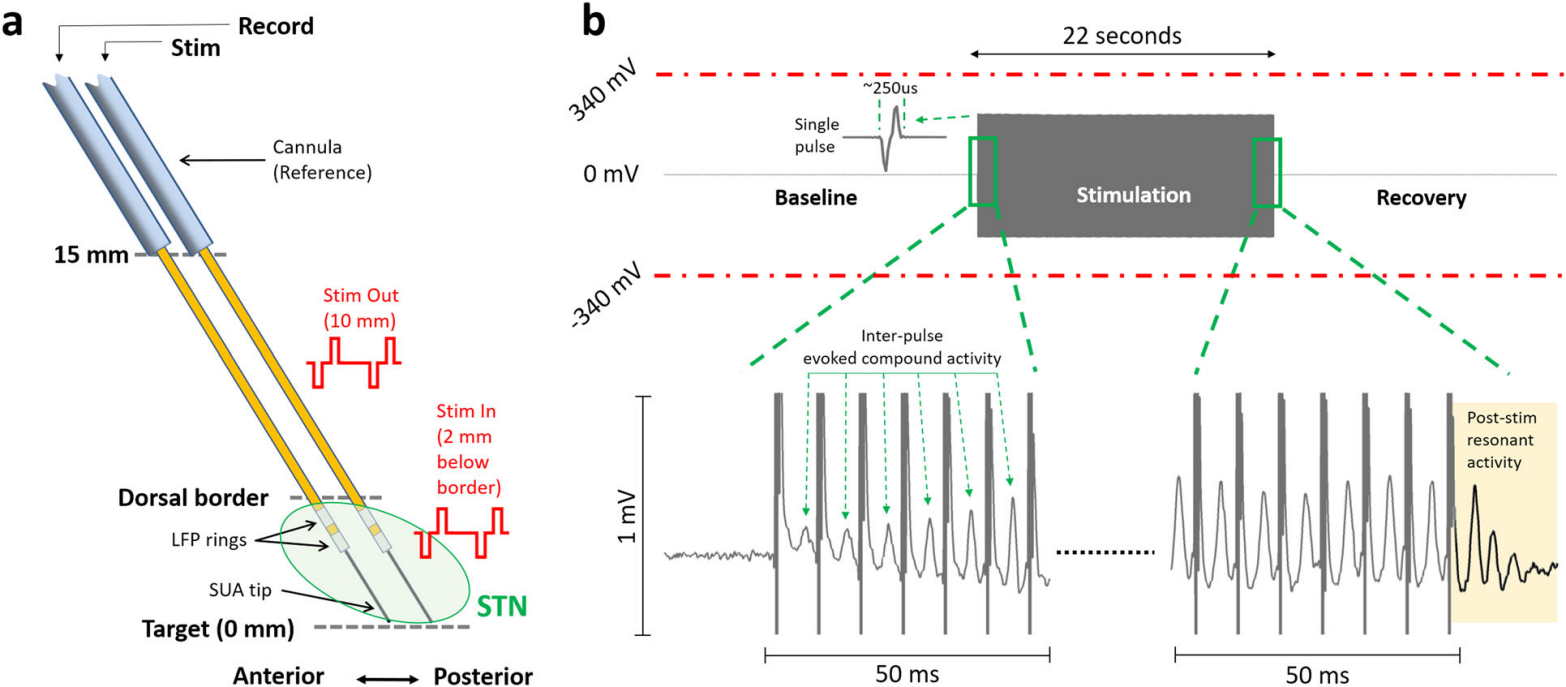
Experimental paradigm and sample stimulation segment showing that LFP recordings did not saturate with the high input range of the amplifier. **a** The microelectrode diagrams depicting the recording and stimulating electrodes. The “out-STN” stimulation was performed 10 mm above the ventral border of the STN. Bipolar microelectrodes with two 0.5-mm wide stainless-steel rings separated by 0.5 mm were used to deliver biphasic electrical stimulation and record LFP activity. The “in-STN” stimulation experiments were performed 2 mm below the dorsal border of the STN, characterized by the increased background activity and neuronal spiking recorded from the fine microelectrode tip. **b** Sample 66 s raw LFP recording illustrates that the amplifier was not saturated during stimulation of the other electrode. Single pulse waveform illustrates that the biphasic stimulation pulse was captured within a short time, allowing the LFP recordings to continue with minimal interruption. Zoomed 50 ms segments from beginning and the end shows evoked potentials induced with each stimulus pulse. The evoked waveform amplitude increases with each pulse and settles after ~10 pulses. With the termination of the stimulation, the resonance in the evoked activity can be observed longer, which dampens within 20 ms.

LFPs were recorded before, during, and after stimulation in 16 STNs at various depths, from two bipolar microelectrodes separated by 2 mm, as depicted in Fig. 1a. Recordings were performed unilaterally from the STN contralateral to the most affected side in ten patients and bilaterally in three patients. The neural recordings started 15 mm above the ventral border of STN (denoted as 0 mm), and the “out-STN” stimulation was performed when the electrodes reached 10 mm. The “in-STN” stimulation experiments were performed 2 mm below the dorsal border of STN. The electrode with the most beta and HFO activity^35,36^ was used for recording the LFPs from the stainless-steel rings situated 3 and 4 mm above the tip, respectively. The LFP rings of the other electrode were used to deliver bipolar, biphasic, cathodic-leading stimulation at 2 mA amplitude and 60 μs pulse width at various frequencies for 22 s. The recorded waveforms were checked for saturation visually and verified to be within the amplitude range of the recording amplifier (Fig. 1b). The amplifier, with its high sampling frequency (38.4 kHz) and large input range (±340 mV), was able to capture the stimulation pulse without saturation and within a short duration (~250 μs, 9–10 samples), followed by evoked LFP activity.

### HFS modulates HFO and evokes resonant compound activity in STN

**Fig. 2:**
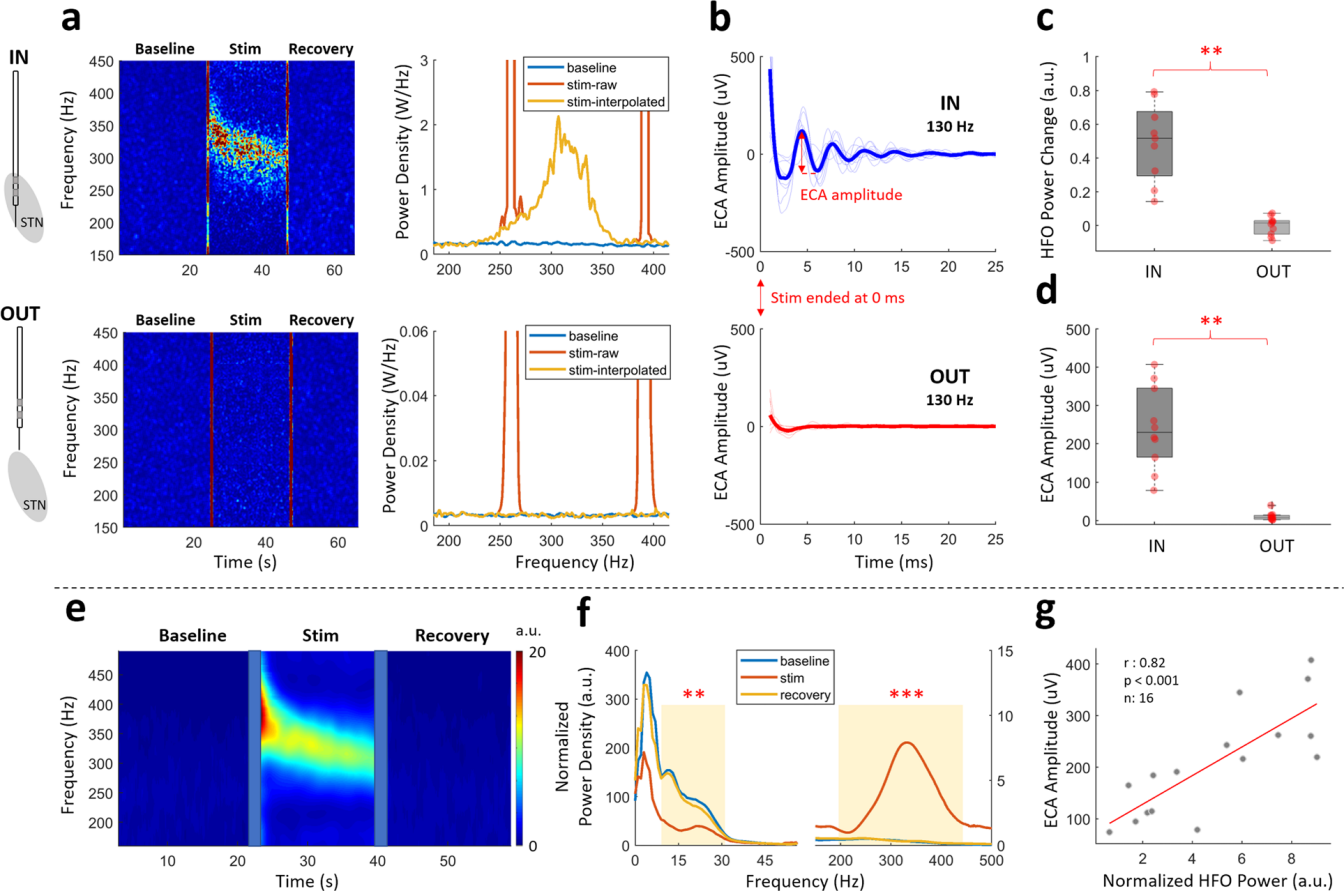
High-frequency oscillations (HFO) and resonant evoked compound activity (ECA) are observed during high-frequency DBS only in the STN. **a** Representative TFM and PSD plots of out- and in-STN stimulation from a sample patient shows HFO was induced only within the STN. The vertical red lines on TFMs are the transition artifacts associated with turning the stimulator on and off. The large artifacts caused by harmonics of the stimulation frequency are interpolated. The color scale of the TFMs is the same as the limits of the *y*-axis of their respective PSD plots. **b** The evoked response waveform at the end of 22 s stimulation was only seen in-STN. The thick lines illustrate the mean waveform for all subjects. The first 1 ms after the stimulation pulse is blanked out due to large artifact amplitude. **c** Comparison of the HFO power change between out- and in-STN stimulations show a significant difference (*n* = 10). **d** Similarly, the difference between ECA amplitude of out- and in-STN stimulations was significant (*n* = 10). **e** The grand average TFM from all 16 hemispheres with 130 Hz in-STN stimulation shows a stark enhancement in the HFO range, similar to the representative subject. The transition artifacts on TFM are masked with blue boxes. **f** The grand average PSD plots for baseline, stimulation, and recovery periods from all 16 hemispheres with high-frequency stimulation in-STN. There was a significant suppression in the beta and significant enhancement in the HFO ranges (*n* = 16). **g** In the STN, the ECA amplitude and induced HFO power correlated (*n* = 16). On each box in the boxplots, the central mark indicates the median, and the bottom and top edges of the box indicate the 25th and 75th percentiles, respectively. The whiskers extend to the most extreme data points not considered outliers, and the outliers are plotted individually using the “+” symbol. The individual data points are also plotted as red circles. ** denotes significance <0.01, *** denotes significance <0.001.

The modulatory effects of therapeutic stimulation were explored with an “OUT vs. IN” STN stimulation paradigm to investigate whether there are STN-specific neural patterns. Figure 2a illustrates the changes in LFP spectrum before, during and after HFS (130 Hz), out- and in-STN for a representative subject. The left panels are the time-frequency maps (TFMs) showing the temporal changes in the LFP spectrum between 150 and 450 Hz range, whereas the right panels represent the average spectral content of baseline and stimulation periods of the corresponding TFMs. The stimulation induced modulations in LFP power between 250 and 350 Hz range in-STN only (Fig. 2a, top row).

The HFS also induced resonant evoked compound activity (ECA) in-STN between pulses (Fig. 1b) as well as at the end of stimulation (Fig. 2b, top) in the form of a damped oscillation. Similarly, lack of observation of ECA out-STN suggests that this response is specific to STN and not a stimulation artifact (Fig. 2b, bottom).

Group analyses show that there was a consistent enhancement around 300 Hz induced by HFS in-STN (Fig. 2a), whereas no modulation was present out-STN with the same stimulation (Fig. 2c, Wilcoxon signed-rank test, *p* < 0.01, *n* = 10). The modulated HFO disappeared immediately after the cessation of the stimulation. The lack of modulation out-STN (Fig. 2a, bottom) suggests that the induced HFO activity is not a stimulation artifact. The amplitude of ECA, calculated as the amplitude difference between the first positive and the first negative peaks, was also significantly higher in-STN (Fig. 2d, Wilcoxon signed-rank test, *p* < 0.01, *n* = 10).

The average TFMs of all 16 hemispheres with 130 Hz stimulation in the STN is shown in Fig. 2e. The enhancement of the HFO band in the group data was similar to that within the representative subject. The peak frequency of HFO started around ~350 Hz and settled towards ~300 Hz towards the end of stimulation (Fig. S1). As the corresponding average spectrum (Fig. 2f) illustrates, there was a significant suppression in the beta band accompanied by a significant enhancement in the HFO range during stimulation, when compared to the baseline (Wilcoxon signed-rank test, *p* = 0.02 for beta, *p* < 0.001 for HFO, *n* = 16). The baseline and recovery sections had very similar spectral characteristics. The beta and HFO bandpowers or their change were not significantly correlated with each other (normalized bandpowers during stimulation: *r* = 0.09, *p* = 0.7; bandpower change compared to the baseline: *r* = 0.04, *p* = 0.8). Interestingly, the enhanced HFO bandpower during stimulation was significantly correlated with ECA amplitude in the STN (Fig. 2g, Spearman, *r* = 0.82, *p* < 0.001, *n* = 16). Although beta band was also suppressed with stimulation, no significant correlation was found with ECA amplitude (*r* = −0.08, *p* = 0.7).

### Low-frequency stimulation does not modulate HFO but induces non-resonant ECA in STN

**Fig. 3:**
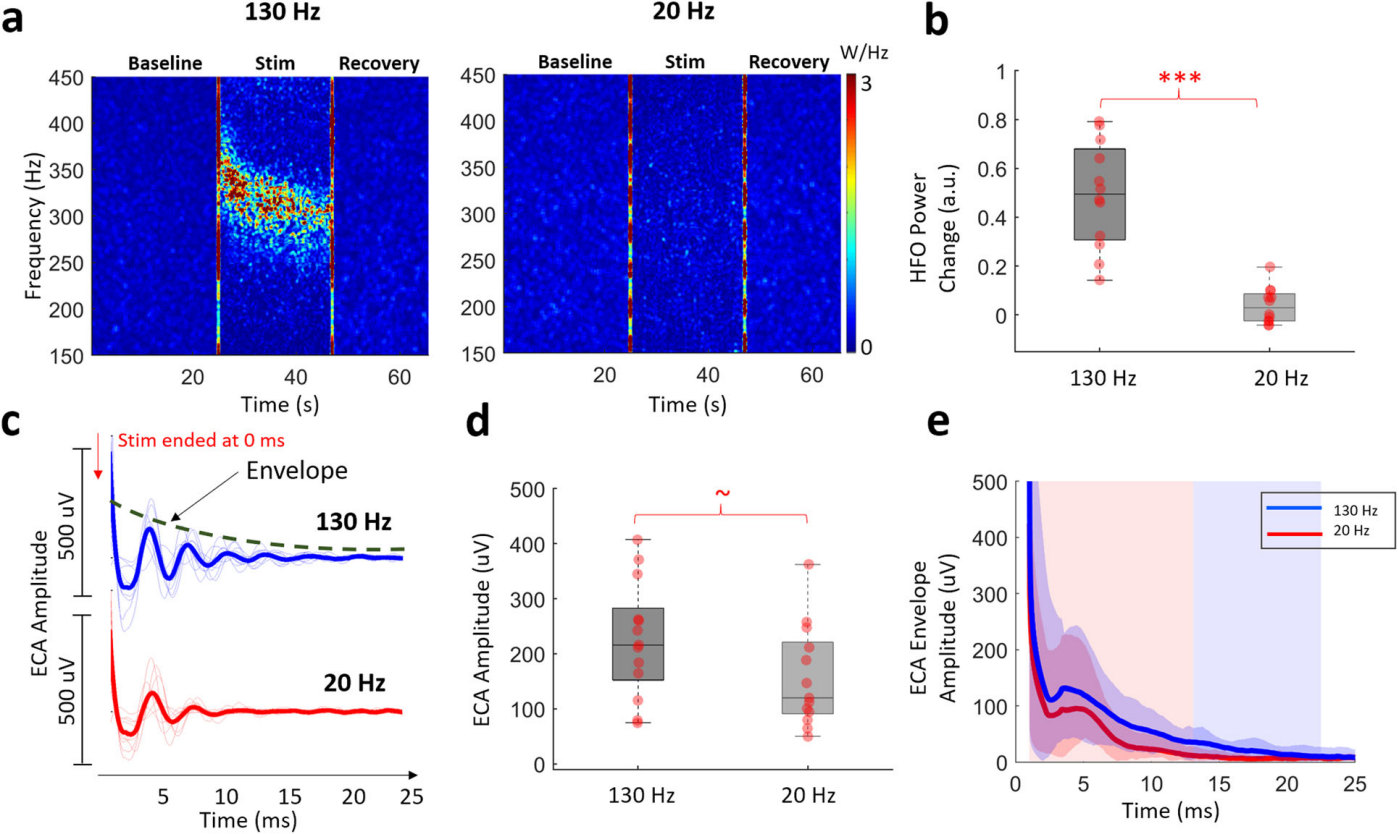
Low-frequency DBS does not induce HFO but evokes compound activity that damped faster. **a** Representative TFMs of high- and low- frequency stimulation from a patient shows that HFO was induced only with the former. The large artifacts caused by harmonics of the stimulation frequency are interpolated. The vertical red lines on TFMs are the transition artifacts associated with turning the stimulator on and off. **b** The HFO power change was significantly higher with high-frequency stimulation (*n* = 13). **c** Both high- and low- frequency stimulation induced ECA. The first 1 ms after the stimulation pulse is omitted due to large artifact amplitude. **d** The difference between ECA amplitude after high- and low-frequency stimulation was only marginally significant (*n* = 13). **e** The damping of ECA derived from the envelope of the Hilbert transform of the waveforms was significantly faster after low-frequency stimulation (mean ± standard deviation: 22.5 ± 5 vs. 13 ± 3.8 ms, *n* = 13). On each box in the boxplots, the central mark indicates the median, and the bottom and top edges of the box indicate the 25th and 75th percentiles, respectively. The whiskers extend to the most extreme data points not considered outliers, and the outliers are plotted individually using the “+” symbol. The individual data points are also plotted as red circles. ~ denotes *p* < 0.06, *** denotes significance <0.001.

To determine whether the HFO and ECA were specific indicators of therapeutic stimulation, we also delivered low-frequency 20 Hz stimulation in-STN. Figure 3a illustrates representative TFMs from before, during, and after high- and low-frequency stimulation, respectively, in a representative patient. The modulation observed in the HFO range with 130 Hz stimulation was not present with 20 Hz stimulation. Group statistics revealed that the power of the induced HFO activity was significantly different between 20 and 130 Hz (Fig. 3b, Wilcoxon signed-rank test, *p* < 0.001, *n* = 13). One can argue that the bandlimited HFO does not appear due to long inter-pulse interval during 20 Hz stimulation. Shortening this distance did not induce the bandlimited HFO activity in a representative patient, which suggest that the lack of bandlimited HFO is not simply due to the long inter-pulse interval during 20 Hz stimulation (Fig. S2).

Despite the lack of modulation in the LFP spectrum in the HFO range, interestingly, the ECA was still present with low-frequency stimulation (Fig. 3c). The amplitude of the 20 Hz ECA was slightly smaller than that of the 130 Hz and the difference was only marginally significant (Fig. 3d, Wilcoxon signed-rank test, *p* = 0.057, *n* = 13). However, 20 Hz-induced activity was not as resonant as 130 Hz stimulation and damped earlier, as illustrated in Fig. 3c, e (mean ± standard deviation: 22.5 ± 5 vs. 13 ± 3.8 ms, Wilcoxon signed-rank test, *p* < 0.001, *n* = 13).

### HFO power is independent from ECA observed between stimulation pulses

**Fig. 4:**
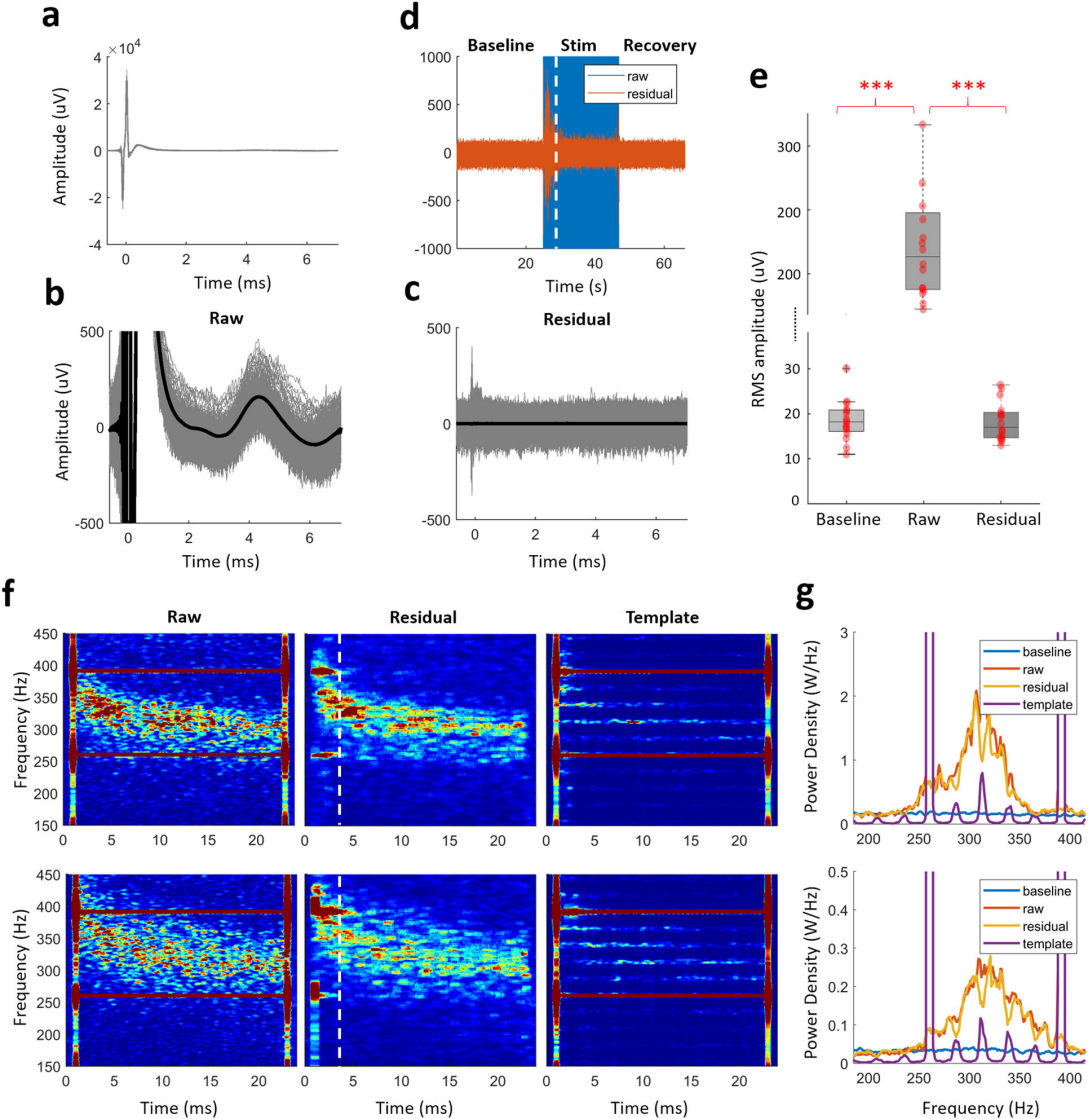
HFO induced by therapeutic 130 Hz DBS is present even after removal of evoked waveform between stimulation pulses. **a** The overlay plot of raw data aligned with respect to the stimulus pulses (0.5 ms pre-onset was used to avoid edge artifacts). The large biphasic stimulation artifact at 0 ms masks both ECA and other LFP activity. **b**, **c** The same segment in **a** shown with 1 mV amplitude scale before and after denoising, to better demonstrate the removal of ECA waveform between pulses (thicker lines indicate the average waveform). The residual was obtained by subtraction of reconstructed segments from the raw data and it is devoid of stimulus pulse and inter-pulse evoked activity. **d** The amplitude range of the denoised (residual) data is similar to the baseline levels, except the first 3 s seconds of transient period. This segment, denoted with the dashed lines, was removed from overlay plots for clarity and excluded from spectral plots as well. **e** The root-mean-square (RMS) amplitude of the baseline segment before stimulation as well as raw and residual traces during stimulation. There was no significant difference between baseline and the denoised stimulation segment (*n* = 16). The first 1 ms including the large stimulation artifact was omitted from the RMS calculations to capture the amplitude levels associated with the evoked response. **f** TFM of the raw and residual segments and the template trace that was removed, as well as **g** their PSD plots from two representative patients illustrate that the denoising primarily removes the artifacts at the sub-harmonics of stimulation frequency while keeping the enhanced HFO intact. The varying power levels of the sub-harmonics throughout the spectrum are due to the leakage from the background activity to the extracted template trace and do not affect the overall peak and power characteristics of the HFO activity when removed. The color scale of the TFMs is same as limits of *y*-axis of their corresponding PSD plots. The dashed lines on TFMs denote the transition artifacts associated with turning the stimulator on and off. On each box in the boxplots, the central mark indicates the median, and the bottom and top edges of the box indicate the 25th and 75th percentiles, respectively. The whiskers extend to the most extreme data points not considered outliers, and the outliers are plotted individually using the “+” symbol. The individual data points are also plotted as red circles. *** denotes significance <0.001.

The correlation observed between induced HFO bandpower and the amplitude of evoked resonant compound activity with HFS was relatively high (Fig. 2g, *r* = 0.82, *p* < 0.001). Naturally, the HFO could be an epiphenomenon of the ECA between stimulation pulses. To test whether these two phenomena are dependent, we executed an adaptive denoising process to remove the inter-pulse ECA as well as the large amplitude stimulation artifacts from the LFP data. A moving average template removal filter (see “Methods” section for the details) was applied to the raw data, in order to isolate and remove the evoked peaks during stimulation. Figure 4a illustrates the representative raw LFP data segmented around the stimulation pulses. The large biphasic stimulation pulse artifact at 0 s is much larger in amplitude in comparison to ECA and typical LFP oscillations. Figure 4b shows the same segments with 1 mV amplitude range, revealing the ECA waveforms between stimulation pulses (Fig. 1b). The templates of ECA waveform following each stimulation pulse as well as the stimulation artifact is reconstructed from these segments. The residual data (reconstructed signal subtracted from raw signal, Fig. 4c) were considered as denoised LFP. The denoising process removed the large stimulation artifact as well as the ECA observed between pulses. Figure 4d demonstrates that the denoising achieved amplitude ranges comparable to the baseline segment before and after the stimulation, except the short 3 s transient period in the beginning which was excluded from further analysis. Figure 4e compares the root-mean-square (RMS) amplitude of baseline segment with that of raw and residual segments in all hemispheres. While baseline and residual segments were not statistically different, the raw stimulation segment was significantly larger in amplitude (Friedman’s test, *p* < 0.001, Tukey–Kramer test, *p* < 0.001, *n* = 16). The first 1 ms which involves the large stimulation artifact (Fig. 4a) was removed from the RMS amplitude calculations to prevent the masking of evoked activity by the stimulation artifact. Figure 4f, g shows the TFMs and the corresponding spectra from two representative subjects during stimulation (between the dashed lines and the last stimulation pulse). The denoising process suppressed the large spectral artifacts at the harmonics of stimulation frequency. Although the ECA has been removed from the raw data (Fig. 4c), it did not change the spectral content of the LFP in the HFO range as much and the correlation between ECA amplitude and HFO power from the denoised traces remained the same (Spearman, *r* = 0.82, *p* < 0.001, *n* = 16), suggesting that HFS-induced ECA and HFO power are independent. Further investigation of a possible relationship between ECA waveform and HFO activity was conducted by modeling a second-order LTI system with the impulse response of a damped oscillation. Analysis with figures available in the Supplementary Section: “Simulations with 2nd order LTI system - damped oscillator” and the Matlab script to generate the simulations provided in this section is provided as a supplementary code file. Although the ECA waveform was replicated, there was no bandlimited HFO activity as in Fig. 4f, g.

### Frequency-dependent modulation of ECA and HFO activity

**Fig. 5:**
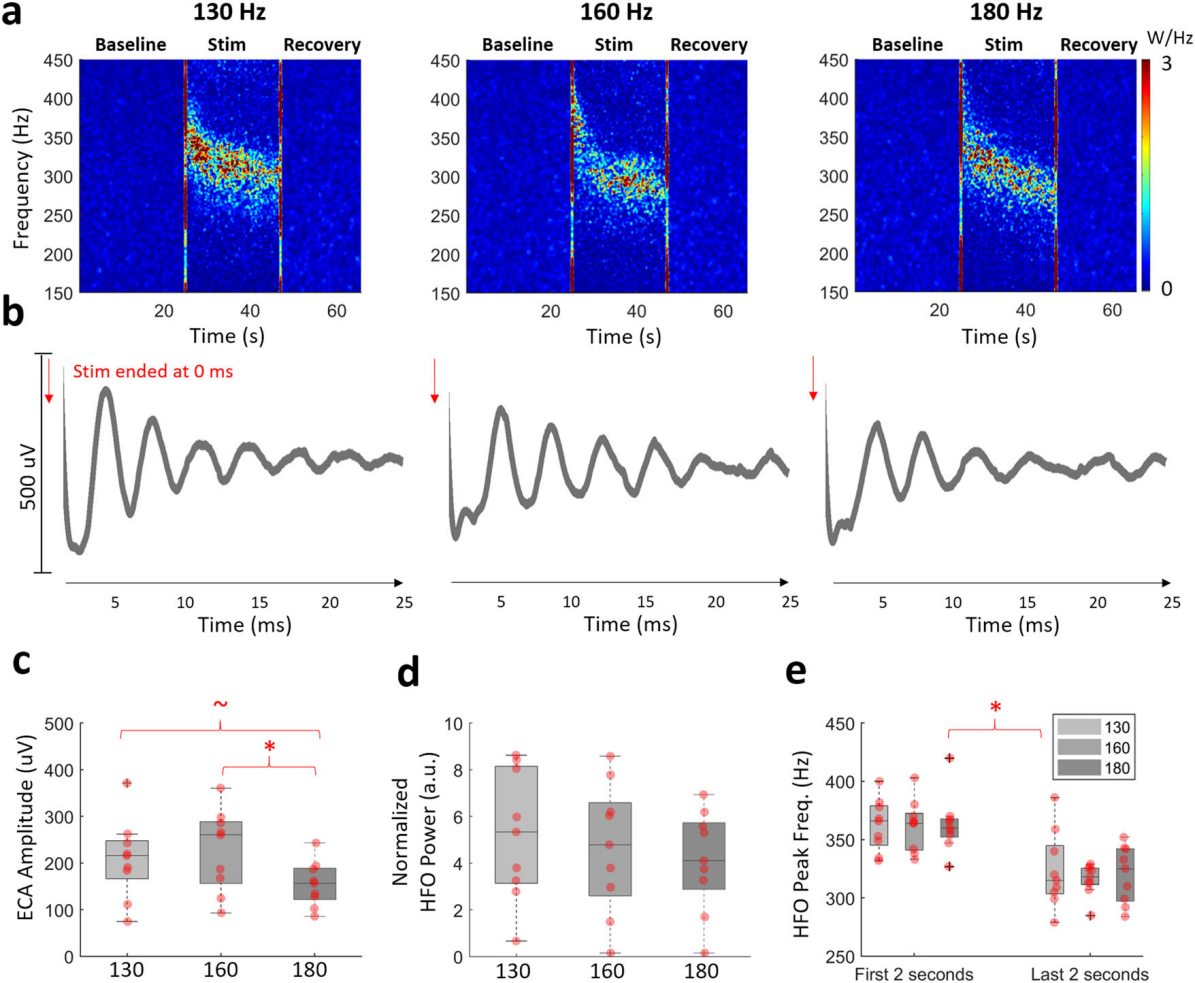
High-frequency stimulations modulate HFO and ECA in different amplitudes. **a** The HFO was enhanced with all stimulation frequencies. The large artifacts caused by harmonics of the stimulation frequency are interpolated. The vertical red lines on TFMs are the transition artifacts associated with turning the stimulator on and off. **b** The ECA waveform after 22 s stimulation period was resonant after all high-frequency stimulations. The first 1 ms after the stimulation pulse is omitted due to large artifact amplitude. **c** The comparison ECA amplitude after 22 s of stimulation showed that ECA after 180 Hz stimulation was slightly smaller (*n* = 9). **d** The induced HFO bandpowers showed a downward trend as the stimulation frequency increased, but the difference between groups was not significant. **e** The peak frequency was significantly higher in the first two seconds compared to last two, but there was no difference between groups based on stimulation frequency. On each box in the boxplots, the central mark indicates the median, and the bottom and top edges of the box indicate the 25th and 75th percentiles, respectively. The whiskers extend to the most extreme data points not considered outliers, and the outliers are plotted individually using the “+” symbol. The individual data points are also plotted as red circles. ~ denotes *p* value <0.09, * denotes significance <0.05.

The dependency of ECA and HFO activity on the stimulation frequency was tested by stimulation of STN with 130, 160, and 180 Hz. Figure 5a shows that the enhancement in the HFO band was visible during all 130, 160, and 180 Hz DBS in a representative subject. The representative evoked waveforms at the end of stimulation in Fig. 5b also show that all three stimulations induced resonance in ECA. We further investigated population response of these metrics. The envelope decay analysis used in Fig. 3e was utilized to quantify resonance in ECA across these three frequencies and despite a decreasing trend as the stimulation frequency increased, there was no significant difference (Friedman’s test, *p* = 0.45, *n* = 9) between three groups in terms of damping durations (Fig. S3, ECA duration after 130, 160, and 180 Hz stimulation: 21.2 ± 4.4, 18.9 ± 3.2, 18.6 ± 2.7 ms respectively). Figure 5c illustrates that the ECA amplitude was slightly smaller with 180 Hz stimulation when compared to the others (Friedman’s test, *p* = 0.01, Tukey–Kramer test 130 vs. 180 Hz, *p* = 0.086, 160 vs. 180 Hz, *p* = 0.013, *n* = 9), which were not significantly different. Although all HFSs induced HFOs and the HFO power appeared to diminish with increased stimulation frequency, the differences among groups was not significantly different (Fig. 5d, Friedman’s test, *p* = 0.46, *n* = 9). The level of beta suppression was not significantly different between groups either (Friedman’s test, *p* = 0.46, *n* = 9). Similar to Fig. 2e, the center frequency of HFO was higher residing between 350 and 450 Hz range during the initial phase of the stimulation and later it decreased, settling around 300–350 Hz (Fig. S1). While the peak frequency was significantly higher in the first two seconds compared to last two, there was no difference between groups based on stimulation frequency (Fig. 5e, Friedman’s test, *p* < 0.001, Tukey–Kramer post hoc, test *p* < 0.05, *n* = 9). Finally, we correlated the HFO enhancement with ECA amplitude for patients with multi-frequency therapeutic stimulation. There was still a significant correlation of 0.77 (Spearman, *p* < 0.01, *n* = 27) between ECA amplitude and HFO bandpower induced by all HFSs.

**Fig. 6:**
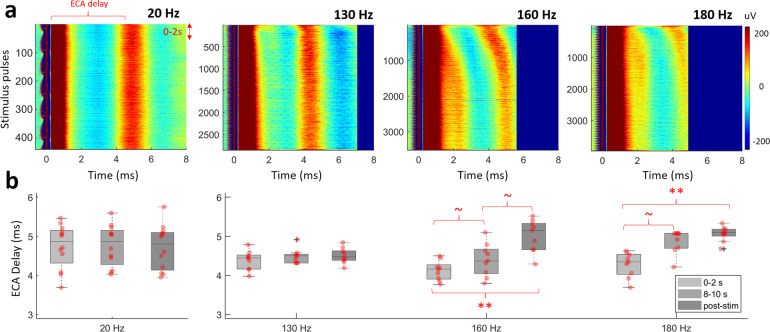
Inter-pulse evoked activity shows adaptation only with high-frequency stimulation. **a** The representative aligned raw data with respect to the stimulus pulse for 130, 160, 180, and 20 Hz stimulation (0.5 ms pre-onset, up to 8 ms is shown). **b** The ECA delay comparison in the 0–2, 8–10, and after 22 s demonstrate the differences in adaptation between 130, 160, 180 (*n* = 9), and 20 Hz (*n* = 13) stimulations. The ECA delay, which denotes the delay of the first evoked peak, was consistent throughout the 20 Hz stimulation period, indicating no adaptation. The fastest adaptation was with 130 Hz, settling after 2 s. On each box in the boxplots, the central mark indicates the median, and the bottom and top edges of the box indicate the 25th and 75th percentiles, respectively. The whiskers extend to the most extreme data points not considered outliers, and the outliers are plotted individually using the “+” symbol. The individual data points are also plotted as red circles. ~ denotes *p* value <0.09, ** denotes significance <0.01.

Despite lack of difference in HFO peak frequency during 130, 160, 180 Hz stimulation and ECA morphology after it, the inter-pulse evoked waveforms varied between these groups. Figure 6a illustrates aligned inter-pulse evoked activity over 22 s of stimulation with various frequencies in a representative patient. While HFS caused an adaptation in the timing of first evoked peak, this was not observed in low-frequency stimulation. Figure 6b quantifies the adaptation by comparing the delay of the first peak after stimulus pulse at the time periods of 0–2, 8–10, and post-stimulation. In all, 8–10 s point was selected since after 10 s, ECA peak in 180 Hz was out of range. As illustrated in the representative subject as well, 130 Hz stimulation settled very quickly (Friedman’s test, *p* = 0.12, *n* = 9) compared to 160 and 180 Hz stimulation (Friedman’s test, *p* < 0.001, Tukey–Kramer post hoc test, *p* < 0.01 for 0–2 s vs. post-stim, *p* < 0.09 for 0–2 s vs. 9–10 s, *n* = 9). With 20 Hz stimulation, the ECA delay between pulses was stable throughout the stimulation period (Friedman’s test, *p* = 0.25, *n* = 13). We further confirmed such adaptations in ECA amplitude and phase with simulations (see Figs. S9–S11).

### HFO power favors specific phases of ECA waveform

**Fig. 7:**
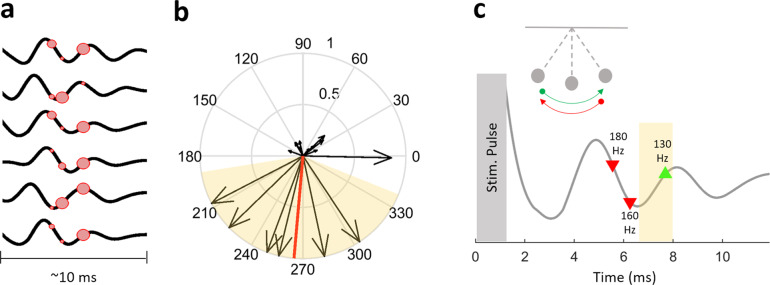
The DBS can be tuned to provide maximum modulatory effect based on the phase of ECA waveform. **a** The ECA waveforms after 22 s of low-frequency stimulation from the six subjects with both 20 Hz and high-frequency stimulation experiments. The location of the red circles denotes where the next 130, 160, and 180 Hz stimulation pulses would be, if the stimulation continued with the specified frequency. The size of the circle denotes the relative amplitude of the induced HFO by that stimulation. **b** The information in **a** is presented on polar coordinates to illustrate the phase preference. The arrow length denotes the relative amplitude of the induced HFO power. The mean angle was 265° and is denoted by the red line. The preferred phase was between 189° and 338° (*n* = 6) and is denoted by the highlighted background. **c** A pendulum and a representative ECA waveform to illustrate the swing-like behavior of the evoked response. The shaded region illustrates the preferred phase angles from panel **b**. The timings of 130, 160, and 180 Hz stimulus pulses are marked as well. When the next stimulation pulse is at a preferred phase location (forward motion, green), the modulatory effects can be enhanced. On the other hand, stimulating at the other phase (backward motion, red) could hinder effect of DBS.

In an effort to explain why HFO was modulated differently with 130, 160, 180 Hz stimulation, we investigated whether the timing of stimulation pulse and the ECA phase affects the modulated HFO power. We utilized the phase of ECA waveform during 20 Hz stimulation, due to its stationary behavior and undistorted phase space. The consistent delay throughout the 20 Hz stimulation period, as seen in Fig. 6, assures that the ECA is not enforced to adaptation in amplitude or phase due to the long duration between consecutive pulses in which the system completely dampens (see also simulations S8D, S9D, S10D, and S11D). The high sampling frequency of the recording amplifier allowing for a more accurate estimation of the phase, which could have been problematic with conventional sampling rates for LFPs (~2 kHz). Figure 7a shows the ECA waveforms after 22 s of low-frequency stimulation from six patients who had a 20-Hz stimulation experiment in addition to 130, 160, and 180 Hz. The location of the red circles denotes where each 130, 160, and 180 Hz stimulation pulse would appear if they were the stimulation frequency. The size of the circle corresponds to the relative HFO bandpower that stimulation induced in each patient. The instantaneous phase information is extracted using Hilbert transform, and represented with arrow with a length corresponding to the relative strength of the induced HFO activity (Fig. 7b). The circular one sample test for mean angle^37^ show that the preferred phase was between 189° and 338° (circ_mtest, *p* < 0.01, *n* = 6). This behavior could be likened to a pendulum or a children’s swing, where the push needs to be made at the right time or phase for the increased effect (Fig. 7c).

## Discussion

To investigate the mechanisms of electroceutical therapy in PD, we have studied the oscillatory neural dynamics of STN-LFPs intraoperatively during low and HFS. We observed that HFS at >100 Hz induced HFOs in 300–450 Hz range as well as an independent evoked resonant compound activity. When DBS was delivered at a non-therapeutic low frequency at 20 Hz, the HFO did not modulate, and the ECA was not as resonant. We also observed that despite having similar modulations, HFO power varies with the therapeutic DBS at different frequencies (130, 160, and 180 Hz), depending on the ECA phase. Our findings suggest that DBS may exert its therapeutic effect by bringing neuronal populations to a “healthy” or “treated” oscillatory state, and the stimulation frequency to maximize these oscillations can be inferred from ECA waveforms.

### Modulated HFO is not an artifact and is not a spectral byproduct of ECA

The relatively high stimulation voltage (V range) and subsequent artifact in the recording systems have historically prevented the exploration of LFPs (μV range), especially in the HFO band due to overlapping artifact harmonics. Therefore, in addition to recording LFPs with an amplifier with large input amplitude range (±340 mV) and relatively high sampling frequency (38.4 kHz), we performed the stimulation experiments in- and out-STN to establish that observed neural modulations are not due to stimulus or saturation artifacts (Fig. 1a). We also ensured that the assessed waveforms during stimulation were within the dynamic recording range of our amplifier (Fig. 1b), which is crucial for the reliability of observed patterns. While HFO was induced only with high-frequency therapeutic DBS (Fig. 2a, c, e, f), evoked activity was present with both therapeutic and non-therapeutic DBS (Fig. 3c, d) only during in-STN stimulation. There was neither evoked response during out-STN stimulation experiments nor HFO was induced (Fig. 2a–d), indicating that both HFO and evoked activity are physiological responses, not mere artifacts.

There was a high correlation between ECA amplitude and HFO power (*r* = 0.82; Fig. 2g), which implied that one could be an epiphenomenon of the other. Later, the dependence of HFO and evoked resonant activity was tested following a denoising process. Specifically, after employing a temporal template extraction filter, we reconstructed the DBS artifact at each pulse and the associated evoked response between pulses, devoid of any LFP activity. This reconstruction was then subtracted from the raw signal to obtain the denoised (residual) data (Fig. 4c). When the spectral content of the raw and residual data was compared (Fig. 4f, g), we observed almost no change in the HFO power. Even though both the DBS pulse artifacts and the evoked waveform were substantially eliminated, the high correlation between ECA amplitude and HFO power was sustained. Furthermore, although we observed an ECA with 20 Hz stimulation at similar amplitude levels (Fig. 6a), there was no simultaneous HFO enhancement (Fig. 3a and Fig. S2). Finally, we have demonstrated through simulation of a damped oscillator similar to the ECA waveform observed in our patients, the presence of ECA itself does not induce the bandlimited HFO activity (Fig. S6) that we observed in vivo (Figs. 2 and 4 and Fig.  S7). Consequently, the existence of HFO activity with HFS even after the removal of the evoked response and observation of evoked activity without HFO during low-frequency stimulation suggest that HFO modulation is independent of ECA and not an epiphenomenon or a spectral artifact/byproduct.

### Resonant vs. non-resonant evoked activity

ECA has been reported in various structures of the nervous system, such as hippocampus^38^, thalamus^39^, spinal cord^40^, cortex^41^ as well as the pallidum^42^ and the STN^42–46^ of PD patients. Specifically, it was observed that STN-DBS causes evoked response in other structures such as cortex^47,48^ and both internal and external parts of globus pallidus^42,49^. In PD-STN, it was shown that the ECA during therapeutic DBS can be used to identify clinically beneficial amplitudes^43^. Using a custom stimulation scheme, Sinclair et al.^44^ recently has demonstrated that evoked resonant activity can also be used to locate the most beneficial stimulation site. We also observed a highly resonant ECA during high-frequency DBS (Fig. 2b). Additionally, we studied stimulation at different frequencies and found that the evoked response exists in the STN with 20, 130, 160, and 180 Hz (Figs. 3c and 5b). While both high- and low-frequency stimulation produced similarly strong ECA (Fig. 3d), the response was significantly more resonant with HFS and lasted longer after cessation of DBS (Figs. 3e, 22.5 vs. 13 ms). When comparing multiple HFS, although 130 Hz was more resonant, the duration of dampening was not significantly different (Fig. S3, 21.2, 18.9, and 18.6 ms for 130, 160, and 180 Hz, respectively).

We speculate that the ECA induced with various stimulation frequencies can be due to the propagation of activity to other structures through projections forming loops^42,50^. Previously, a stereotypical periodic pattern of neuronal responses in globus pallidus (both interna and externa), putamen, and cortex were reported that were same at low- (50 Hz) and high-frequency (100 and 130 Hz) STN-DBS immediately consequent to the DBS pulse^50^. This periodic form of activity has returned to the baseline after ~8 ms not only in these structures but also in STN^6^. Since STN sits in a highly interconnected cortico-basal ganglia-thalamo-cortical network, stimulus at high frequency might have induced ECA in an underdamped oscillatory form through the feedback loops^1,42,51,52^. Additionally, given that ECA can even be recorded from the spinal cord^40^, it is likely that the ECA represents the response of a larger network and not necessarily a local circuit.

The reason for the longer lasting resonance with HFS can be explained with a pendulum/swing analogy (Fig. 7c). If each stimulus pulse is the force pushing the pendulum (i.e., the network) in one direction, with low-frequency stimulation, there is a longer period between each push (i.e. stimulus pulse) and that this duration is long enough for the pendulum to reach equilibrium. Therefore, we observe a steady response in ECA morphology over 22 s of stimulation for 20 Hz (Fig. 6 and Figs. S8–S11) as the response completely dampens before the next pulse hits the system. However, with frequent hits not allowing the pendulum to come to a stop, HFS starts to activate a larger pool of neurons or a network yielding resonance by injecting sufficient paced energy to the system^53–55^. Our in vivo observations and simulations (Figs. S9–S11) provide electrophysiological evidence for the “Resonance and Carrier Signal Effect” hypothesis of Montgomery and colleagues^50,56,57^, which proposes that the resonant ECA is the form of amplified periodic responses to the consecutive stimulation pulses, in accordance with the definition of resonance is physics.

The adaptation on the inter-pulse evoked response (Fig. 6) shows that the network reaches a steady state after an initial ramp-up period (3–10 s) with stimulation at high frequencies and in particular a longer delay at 160 and 180 Hz (Fig. 6b). In that sense, therapeutic DBS likely entrains the network^58^ to its steady state over several seconds based on the consistence of the response from the intrinsic oscillators in the system to the first and consecutive future pulses^56,59^. While there are likely multiple oscillators within the basal ganglia-thalamo-cortical system, it has been suggested that the main or average frequency is approximately 130 Hz^50,56^. Schmidt et al.^42^ and Wiest et al.^46^ have also shown the temporal adaptations of ECA waveforms over longer durations (>1 min), mainly at 130 Hz stimulation, in line with our observations. Recently, Schmidt et al.^42^ has also studied ECA after multiple low-frequency stimulations and reported no change over time, as we have shown in Fig. 6.

It is also possible that resonance in ECA is due to total injected energy^53,54^, similar to a large push to the pendulum causing a longer lasting swing. In that case, even 20 Hz stimulation with high amplitude might cause a longer lasting evoked response. However, an animal study reported that even at high amplitudes, 20 Hz stimulation did not pace the subthalamic neurons, as it did during >100 Hz stimulation^60^. The same group also reported that the pulses must be close enough to one another to override the deleterious STN activity^61^. Both our in vivo (Fig. 3) and simulation experiments (Fig. S6) demonstrate that the long inter-pulse interval allows the system to reset and thus the compound effect is not observed with low-frequency stimulation.

### Modulation of the multiscale neural activity with dopaminergic medication and therapeutic DBS

We observed HFO activity initially starting at 350–400 Hz range and later settling around 300 Hz during HFS of STN (Figs. 2e and 5a, e and Fig. S1). There is an abundance of reports regarding the modulation of ~300 Hz HFO in the STN of PD patients undergoing dopaminergic therapy^29–33^. The HFO activity appears concurrently with the therapeutic effect of the medication, representing the “ON state”^29,31,32^. This rhythm was suggested to be a “coordinating clock” that paces the neural excitability^31^. Indeed, it has been proposed that the bidirectional connection between STN and the external segment of the globus pallidus is well-positioned to form a “central pacemaker” in the basal ganglia^62–64^. The physiological ~3 ms synaptic transmission delay between these two structures^10,65^ could explain the genesis of ~300 Hz HFOs as an indication of functioning pacemaker. Although there are no reports of LFP activity in the healthy human STN, similar HFO activity centered around 300 Hz has been observed in the healthy non-human primates and disappeared after 1-methyl-4-phenyl-1,2,3,6-tetrahydropyridine (MPTP) treatment inducing PD (see Fig. 1 in ref. ^66^). The lack of 300 Hz HFO activity or deviation from it in STN could be a marker of disease state, and high-frequency DBS might be restoring the HFOs to their healthy state similar to pharmacological treatment^29,30,32,33^. There is further evidence that nonlinear features extracted from HFOs correlated with improvements of motor function in patients with PD proposing that HFOs might contain vital information about the disease state or its severity^29–33^.

At a finer scale, earlier studies on non-human primates reported an increase in bursting neuronal activity and instantaneous firing rates after MPTP treatment^67,68^, suggesting that the emergence of bursting firing is a marker of PD state. Later, STN-DBS at 130 Hz has been reported to restore motor function in MPTP-treated primates and 6-hydroxydopamine (6-OHDA)-treated rats by inducing more regular firing patterns, higher average firing rates, and lower burstiness^6,10,50,69,70^. These observations in the form of the migration from bursting to regular firing due to high-frequency DBS were further confirmed with a computational model of the cortico-basal ganglia-thalamo-cortical loop in both normal and parkinsonian conditions^18^. Since single neuronal recordings are not common in PD patients in the medicated state, not much was known regarding the neuronal firing patterns in STN modulated with dopaminergic therapy. Our recent case report investigating the firing patterns of individual neurons and HFO activity in STN intraoperatively has provided a unique opportunity to observe the multiscale neural activity in a PD patient after dopaminergic treatment state^71^. We reported that the STN was dominated with 300 Hz HFO activity and there were no pathological burst-type firings in the medicated state. In this subject, unlike recordings from PD patients in the unmedicated state^72,73^, tonic (regular) spiking was observed together with ~300 Hz HFOs instead of bursts-type firings, with results matching the experimental observation of the regularizing effects of therapeutic STN-DBS on firing rates of STN^11,60^ and other basal ganglia structures^9,10,58,74^. Although it was a single case, the ~300 Hz HFO and regular/tonic neuronal firings in STN with dopaminergic medication^71^ resemble to the regular spiking activity reported previously^9–11^ and the 300–400 Hz oscillatory response presented here during high-frequency DBS, suggesting that the mechanisms of both therapies might be similar as well. It was proposed that DBS therapy overrides the pathological burst-type firings with a stimulus-induced regular (tonic) pattern^9^. Interestingly, earlier computational simulations on information processing through neuronal circuits also suggested that low frequency and irregular spiking activity has an impairing effect on information processing while high frequency and regular activity is the least deleterious^16,75^.

It is important to note that a contradictory hypothesis was suggested by Sinclair et al.^45^, since the authors report a decrease in HFO frequency after high-frequency DBS, compared to the baseline. Therefore, the authors suggested that DBS and dopaminergic therapy have different mechanisms of action. For the reasons including saturation and large stimulus artifact, past work generally focused on the LFP activity in the lower frequencies during stimulation^9,24^ or following the cessation of stimulation^45,76^ with the presumption that neuronal activity observed immediately after stimulation would be representative of neuronal activity during stimulation. However, it has been shown that, after the application of artifact removal algorithms, what happens after stimulation does not necessarily reflect the changes in the neural activity during DBS^77^. In 16 STNs recorded, we observed a consistent power enhancement in the HFO range around 300 Hz (Fig. 2e, f) during stimulation at different frequencies >100 Hz, indicating that the high-frequency therapeutic DBS may have similar mechanisms to pharmaceutical therapy^78^, which is to drive the basal ganglia circuit into a physiological oscillatory equilibrium^17,31^. Consequently, DBS may function by promoting neural activity similar to the stable electrophysiological state in the basal ganglia^60^.

### Electrophysiological basis for selection of optimal DBS frequency

Clinical observations suggest that HFS (>100 Hz) of STN and pallidum is therapeutic for PD^57,79,80^, whereas low-frequency DBS has does not affect or worsens the symptoms^81,82^. One of the hypotheses of mechanism of DBS is that high-frequency DBS saturates the neuronal response (driving neurons into the refractory period) and creates informational lesions^13,83^. In that case, the higher stimulation frequencies should have resulted in better therapeutic response, due to faster repetition as well as the more energy delivered when applied with same amplitude and pulse width. Yet, the studies exploring the frequency as a parameter for DBS have reported that although >100 Hz stimulation can ameliorate the effects of PD^79,80,84^, ~130 Hz produced the most corrective response. They report, as the stimulation frequency increases further away from 130 Hz, especially after 160 Hz, the clinical benefits starts to decrease^50,80,84^. These experimental observations were further validated with a computational model of the cortico-basal ganglia-thalamo-cortical loop in both normal and parkinsonian conditions^18^. When contrasting various HFSs at 130, 160, and 180 Hz, we observed that the power of induced HFO and ECA amplitude was different (Fig. 5c, d). Specifically, 180 Hz has consistently provided the lowest HFO enhancement and resonant ECA generation. Therefore, the amplitude of ECA waveform and power of HFO band can potentially be used as biomarkers to assess the therapeutic efficacy of DBS.

Although we show the LFP modulations are generally stronger with 130 Hz, this may not be the case in an individual patient. Then, how can one find the most therapeutic frequency for a specific patient? We found that there was a relationship between HFO strength and the instantaneous phase at which each pulse hit the ECA waveform (Fig. 7). Specifically, we noted that a certain portion of the ECA phase space promoted the HFO. The highlighted phase intervals are likely the positions of consecutive stimulation pulses promoting resonance. Overall, these observations indicate that although there is a wide range of frequencies that can be used for HFS, it is likely that only a limited range will be effective in promoting maximum resonance yielding better therapeutic outcomes for an individual subject, depending on their intrinsic physiological oscillatory state. Our simulations with a damped oscillator of different peak frequency and resonance levels demonstrate that depending on the intrinsic oscillator, we can tailor the stimulation frequency for maximum modulatory effect (Fig. S9 favors 130 Hz, Fig. S10 favors 160 Hz, Fig. S11 favors 180 Hz stimulation). These observations are in line with the previous work involving computational simulations^18^ and human subjects^80,84^. Going back to our analogy in Fig. 7c, the ECA phase might correspond to different positions of the pendulum. Thus, although DBS at multiple high frequencies might provide some level of clinical benefit for a patient as shown by previous studies, one could pinpoint the most efficient one by checking the ECA waveform and the power of modulated HFO band. In such a system, one could stimulate the patient with non-therapeutic low-frequency for a short time (to obtain the characteristics of the intrinsic oscillators) and propose a range for the optimal DBS frequency based on the temporal characteristics of the resulting ECA waveform. Current closed-loop DBS paradigms only focus on the amplitude of the stimulation^85,86^. Yet, the adaptation of all parameters is needed for a truly adaptive DBS therapy. Here we presented the frequency optimization, but future studies will be conducted to test whether the induced HFO can be utilized to optimize the other parameters.

### Burden on the beta band

Suppression of exaggerated beta band oscillations in the STN of PD patients with DBS^9,24,87^ and dopaminergic therapy^25,29,31,32,88^ is a well-established phenomenon. Moreover, the beta band has been investigated by many groups as a biomarker for closed-loop DBS^85,86,89,90^. We also observed a significant decrease in beta power with HFS, but there was no correlation with evoked activity amplitude. A compelling argument for the lack of such a relationship could be that the beta band is associated with many healthy functions in the brain, such as its suppression with movement or even planning or imagination of it^91,92^, its modulations with wakefulness^93,94^ and decision-making^95^. Therefore, despite established reports of its modulations in PD, the multifaceted involvement of beta band in physiological processes might affect its performance as a biomarker by itself for the diseased state, especially in the freely behaving patients with a chronic DBS implant^96^. It is likely that, as Foffani et al.^31^ proposed, there might be a high-frequency “clock” that is not directly involved in motor functions, but rather regulating the neural synchrony in order to guarantee the specific modulation of individual actions that are controlled by lower frequencies. Therefore, we speculate that beta band must be—at least—combined with other state specific biomarkers for robust and reliable operation of long-term adaptive stimulation schemes such as closed-loop chronic DBS^96^.

### Limitations and future work

In this study, we have studied LFPs during multiple HFS and at 20 Hz, which has not been reported together before. Yet, due to intraoperative setting of this study, we were constrained by time-related limitations. First, given that the effective DBS frequency ranges from 60 to 185 Hz^84,97^, it is important to scan a broader frequency range with more granular steps. Moreover, we only studied the acute effects of the stimulation since it was 22 s. Others have shown that the adapting modulations of LFP during DBS takes longer than ~1 min^42,46^. The modulating effects of different HFS need to be validated over longer durations for potential chronic applications. Finally, for any definitive claims over therapeutic effect, we need to obtain the blinded clinical data regarding patients’ symptoms. Currently, we only show the clear contrast between HFS and low-frequency stimulation as therapeutic and non-therapeutic. For distinguishing the optimal HFS, our results only provide potential biomarkers, and we postulate potential mechanisms of action with the help of relevant literature. The systematic clinical data will be the gold-standard in validating these biomarkers. Since these are relatively laborious tasks for intraoperative setting, future studies with chronic implants will be needed to accomplish them.

Finally, despite consistency of our observation and satisfactory statistical significance of our results, our sample size (16 STNs, 13 patients) is modest. Although the outcomes of the experiments comparing in-STN vs. out-STN and 20 vs. 130 Hz were highly significant, future studies with more subject will be needed regarding the use of ECA phase–HFO power relationship in closed-loop therapy as well as the establishment of these patterns in other brain structures and neurological disorders.

## Conclusion

In this work, we have presented unique electrophysiological modulations in the STN with therapeutic DBS that shed light onto the mechanisms of the electroceutical therapy in PD. We observed that at high frequencies, stimulation induces HFOs similar to what is reported in healthy primates and PD patients under dopaminergic treatment. More importantly, we observed that the stimulation frequency maximizing these oscillations in individual subjects could be inferred by the phase of ECA waveform, opening the doors for a truly adaptive DBS where not only the amplitude but the frequency of stimulation is also optimized for individuals. Future studies with more subject are warranted regarding the use of ECA phase–HFO power relationship in closed-loop therapy as well as the establishment of these patterns in other brain structures and disorders.

## Methods

### Patients

Thirteen patients (two females) with PD undergoing awake bilateral STN-DBS implantation at Baylor St. Luke’s Medical Center were included in the study. Their ages ranged from 53 to 70 (mean ± standard deviation = 60.5 ± 4.7). Recordings from three patients were obtained bilaterally and the rest of the patients were recorded unilaterally from the hemisphere contralateral to the most affected side, totaling 16 STNs. Detailed patient demographics and the types of experiments performed in each patient are denoted in Table S1. Briefly, the “in- vs. out-STN stimulation” paradigm was executed in ten hemispheres to differentiate the modulatory effects of therapeutic stimulation from potential artifacts and was stopped since the statistical significance was achieved. “Multiple high-frequency stimulation” paradigm was executed beginning with patient 5. Stimulations with 130, 160, and 180 Hz were delivered in a randomized order. However, 20 Hz stimulation was not performed due to time constraints in patients 8–10. The study protocol was approved by the Institutional Review Boards of Baylor College of Medicine and University of Houston. All patients provided written informed consent.

### Surgery and recordings

Patients were requested to stop medication at least 12 h prior to the surgery and all recordings were obtained in the awake state using local anesthesia. The stereotactic coordinates and trajectories to the STN were identified by fusing preoperative magnetic resonance imaging (MRI) and computerized tomography (CT) scans on a neuro-navigational platform (StealthStation S7, Medtronic, Ireland). In each hemisphere, awake recordings were performed to validate the targeting, using a set of two parallel microelectrodes separated by 2 mm (center-to-center) using the 5-cannula BenGun with “+” configuration (Fig. 1a). Additional to the center track, one of the anterior, posterior, lateral, or medial tracks was selected by the neurosurgeon on a patient specific basis through analysis of the preoperative MRI. The bipolar microelectrodes (Microprobes for Life Sciences Inc., MD, USA, Fig. S4) were initially placed at 15 mm above the ventral border of the STN (denoted as 0 mm) and advanced towards the target in 0.5–1 mm steps using NeuroOmega drive (AlphaOmega, Israel) (Fig. 1a). The dorsal border of STN was determined in real time by an experienced neurologist via visual and auditory inspection of the single unit firings from the high-impedance tungsten tip (0.4–0.8 MOhm), per standard clinical protocol. The dorsal STN border was identified with a prominent increase in the background activity and spiking. The LFPs were recorded from the 0.5-mm wide stainless-steel rings (3 and 4 mm above the tip, 3–4 kOhm impedance) located on the shaft of the electrode. The cannula was used as reference. The “out-STN” stimulation was performed when the electrodes reached 10 mm. The “in-STN” stimulation experiments were performed 2 mm below the dorsal border of STN (Fig. 1a). The electrode with the most beta and HFO activity^35,36^ was used for recording the LFPs. The stainless-steel rings of the other electrode were used to deliver bipolar, biphasic, cathodic-leading stimulation at 2 mA amplitude and 60 μs pulse width at various frequencies for 22 s, using the Grapevine Neural Interface Processor (Ripple, UT, USA). The recordings were obtained with the gHiamp bio-amplifier (gTec, Austria) at 38.4 kHz sampling frequency, 24 bit A/D resolution and ±340 mV input range. The data were stored in a computer hard drive for offline processing.

### Signal processing

The data acquisition and stimulation were performed using custom developed Simulink models in Matlab R2014a, and Matlab R2018a (Mathworks, MA) was used for both signal processing and statistical analyses. The raw LFP traces were visualized to ensure the recordings were within the input range of the amplifier (±340 mV). The traces were forward and backward filtered with a second-order Butterworth high-pass filter with 2 Hz cut-off frequency. The spectral analyses were conducted using Thompson multi-taper estimate with four slepian windows for each 1 s of data (with 50% overlap). The large artifacts caused by harmonics of the stimulation frequency were interpolated with 5 Hz width for HFS and with 1.5 Hz width for low-frequency stimulation. The spectra were normalized using the mean activity between 500 and 600 Hz to account for amplitude differences between patients and smoothed with Matlab’s “smoothdata” function (“rloess” method was used with window length of 100) for frequencies over 100 Hz. From the spectrogram, the beta and HFO bandpowers were calculated as mean power between 12–30 and 200–450 Hz, respectively.

Evoked activity waveforms were processed by subtraction of a fitted exponential first to remove the decaying response from the amplifier settling. The waveform was then smoothed with a Savitzky-Golay filter^98^. Matlab’s “smoothdata” function was used with method “sgolay”, window length of 90, and degree of 3. The processing did not affect the morphology or the duration of the evoked activity (Fig. S5). The evoked waveforms were analyzed monopolarly, since the waveform was present on both contacts. To quantify the resonance, the envelope of the ECA was detected using Hilbert transform, and the first 30 ms of the envelope at every sample point was compared to the envelope of 5 ms of baseline activity from the same recording to check if the ECA waveform amplitude is still higher than the baseline levels (one-tailed *t*-test, *p* < 0.05).

Inter-pulse evoked activity was reconstructed from the segmented traces in order to investigate if HFO activity is an artifact caused by the evoked waveform. The peaks of stimulus pulses were detected, and the 22 s of data during each stimulation was aligned with respect to the peak. A moving template was extracted using the Principal Component Analysis method over each 1 s of data with 50% overlap. The largest eigenvector corresponded to the stimulus artifact and accompanying inter-pulse evoked activity. Each waveform in the aligned data was reconstructed in this way and the residual was calculated by subtraction of the reconstructed data from the original raw trace.

### Statistics and reproducibility

The comparative statistical analyses were performed using paired, non-parametric Wilcoxon signed-rank test for two groups, given the non-normal distribution of some variables studied (Anderson-Darling test, *p* < 0.05). For comparison of more than two groups, Friedman’s test was utilized, with Tukey–Kramer test as post hoc method for pairwise comparisons. Spearman coefficient was used for the correlation analyses. The significance of the decay of evoked activity of each individual stimulation was tested with one-tailed *t*-test due to the normality. The threshold alpha level to determine significance was 0.05, unless otherwise noted. The circular statistics regarding phase angles were performed using Circular Statistics Toolbox^37^. On each box in the boxplots, the central mark indicates the median, and the bottom and top edges of the box indicate the 25th and 75th percentiles, respectively. The whiskers extend to the most extreme data points not considered outliers, and the outliers are plotted individually using the “+” symbol. The individual data points are also plotted as red circles. The raw data corresponding to these points are also provided as a spreadsheet in the supplementary data file.

### Reporting summary

Further information on research design is available in the Nature Research Reporting Summary linked to this article.

## Supplementary information

Supplementary Information

Description of Additional Supplementary Files

Supplementary Data 1

Supplementary Code

Reporting Summary

## Data Availability

The data that support the findings of this study are available on request from the corresponding author. The raw data are not publicly available as the data might contain potentially identifying or sensitive information that could compromise the privacy of the research participants.
