# Supplementary material for: Electroceutically induced subthalamic high-frequency oscillations and evoked compound activity may explain the mechanism of therapeutic stimulation in Parkinson’s disease
Source: Commun Biol. 2021 Mar 23;4:393. doi: 10.1038/s42003-021-01915-7 (PMC7988171; doi:10.1038/s42003-021-01915-7)
Supplement: Supplementary file 1 — Supplementary Information [file 42003_2021_1915_MOESM1_ESM.pdf]

Supplementary Information for "Ozturk et al., Electroceutically induced subthalamic high-frequency oscillations and evoked compound activity may explain the mechanism of therapeutic stimulation in Parkinson's disease"

Musa Ozturk, Ashwin Viswanathan, Sameer A. Sheth, Nuri F. Ince

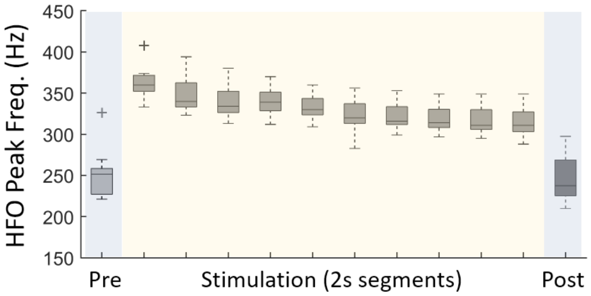

Figure S1: The HFO peak frequency change before, during and after stimulation with high-frequency DBS. The stimulation was split into 2s segments to show the change of frequency over time. The peak frequency was ~250 Hz before stimulation and shifted to between 350-400 Hz immediately with the onset of stimulation. It asymptotically decreased, settled at ~300 Hz after 16-18s and shifted back to ~250 Hz after cessation of stimulation. The peak frequency during pre- and post-stimulation were not significantly different (Wilcoxon signed-rank test, p>0.05, n=27). There was a significant difference in peak frequencies between all 2s consecutive blocks (Wilcoxon signed-rank test, p<0.05, n=27) except the last two, indicating the settlement towards the end of stimulation period. All high-frequency stimulations were combined since there was no difference between their peak frequency over time (Figure 5e). On each box in the boxplots, the central mark indicates the median, and the bottom and top edges of the box indicate the 25th and 75th percentiles, respectively. The whiskers extend to the most extreme data points not considered outliers, and the outliers are plotted individually using the '+' symbol.

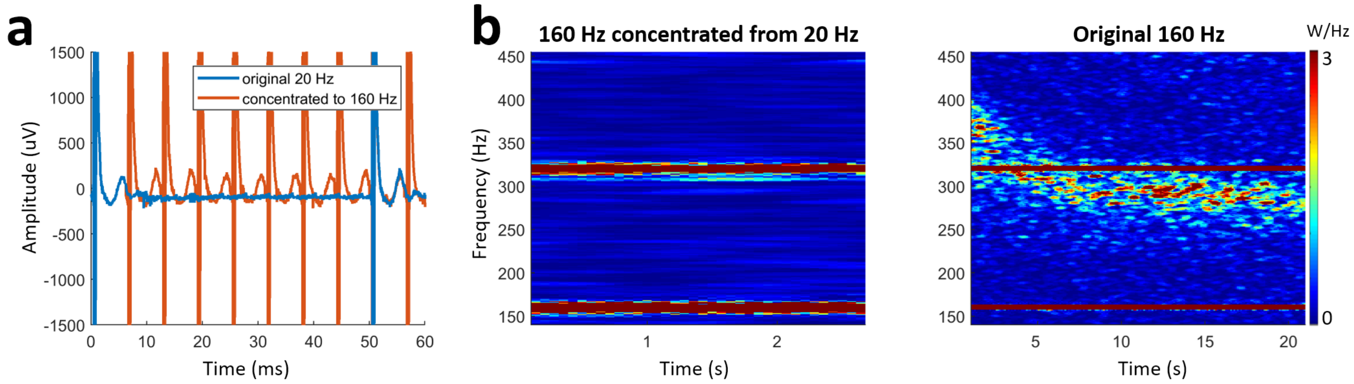

Figure S2: The HFO induced during high-frequency stimulation is not due to inter-pulse evoked activity. (a) The original 20 Hz stimulation segment (blue line) was aligned with respect to the stimulus pulse, trimmed and then concatenated to mimic the 160 Hz stimulation (red line). That is, the 50ms inter-pulse waveform in 20Hz stimulation was shortened to 6.25ms, to match the period of 160 Hz stimulation and concatenated back to effectively obtain a 2.75s segment. (b) The TFM was computed with the concatenated data, which lacked the HFO seen in 160 Hz stimulation of the same patient despite clear repetition of the ECA between pulses. If HFO in the spectrum was caused by repeated ECA waveforms, it would have appeared in the TFM of the concentrated trace presented in B. This analysis was conducted in addition to the template removal approach (Figure 4), to test the dependence of ECA and HFO activity.

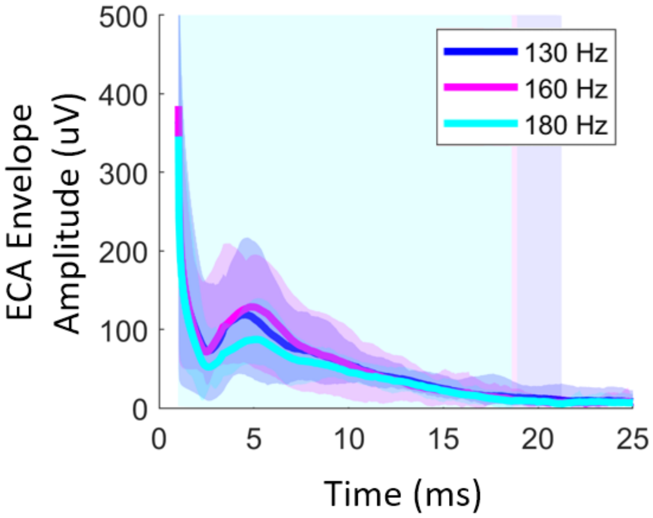

Figure S3: The damping of ECA derived from the envelope of the Hilbert transform of the waveforms takes similar amount of time when comparing different high-frequency stimulations. (ECA duration after 130, 160 and 180 Hz stimulation: 21.2±4.4, 18.9±3.2, 18.6±2.7ms respectively). Although there was a decreasing trend as the stimulation frequency increased, the damping duration was not significantly different between stimulation frequencies (Friedman's test, p=0.45, n=9).

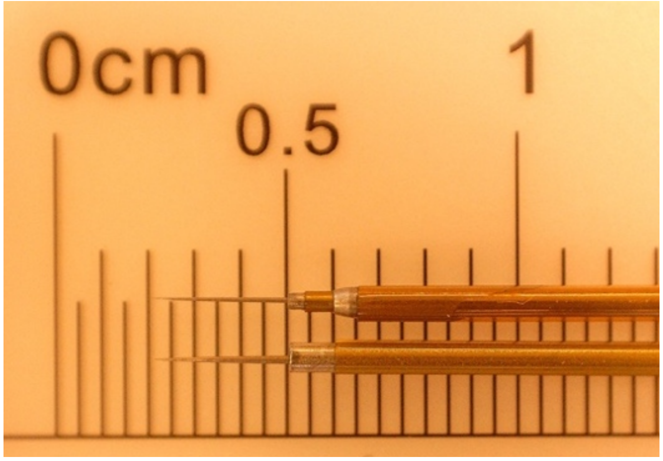

Figure S4: The photograph of bipolar microelectrode (Microprobes for Life Sciences Inc, MD, USA) used during recordings (top). The high-impedance tip (0.6-0.8MOhm) is used for the monitoring of single unit activity for border detection, whereas 0.5mm wide stainless-steel rings situated 3 and 4 mm above the tip were used to record LFPs and deliver stimulation. A monopolar microelectrode (Neuroprobe, AlphaOmega, Israel) is also pictured for comparison (bottom). Both electrodes are identical with their physical dimension such as outer diameter and length. The top microelectrode contains two stainless steel rings to achieve bipolar stimulation and recording.

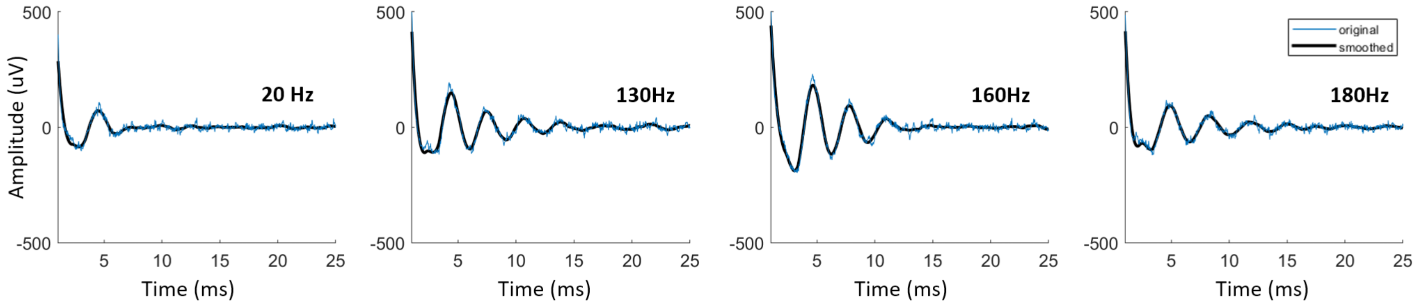

Figure S5: ECA waveforms after 22s second stimulation at different frequencies from a representative STN. For smoothing, MATLAB's smoothdata function with parameters smooth_eca = smoothdata(eca, 'sgolay', 90,'Degree',3) were used. This operation helped with robust peak detection for other analyses and did not change the morphology of the waveforms.

Table S1: Patient demographics and the performed experiments. Phenotype TD refers to tremor dominant, PIGD refers to postural instability and gait disorder and MIX is when the patient shows traits of both groups [1]. UPDRS stands for unified Parkinson's Disease Rating Scale.

Patient Info							Experiments Performed		
Pt	Age	Duration	Gender	Hemisphere	Phenotype	Pre-surgery UPDRS III (Med OFF-ON)	Out-130 Hz	20 and 130 Hz	160 and 180 Hz
1	58	7	F	Left, Right	TD	46-35	x	x	
2	62	17	M	Left, Right	TD	48-38	x	x	
3	53	4	F	Left, Right	TD	43-32	x	x	
4	61	13	M	Right	TD	24-16	x	x	
5	60	11	M	Left	PIGD	35-9	x	x	x
6	57	11	M	Right	TD	20-9	x	x	x
7	69	9	M	Right	TD	59-46	x	x	x
8	61	18	M	Left	MIX	40-10		x*	x
9	70	8	M	Left	TD	45-23		x*	x
10	58	8	M	Left	PIGD	34-17		x*	x
11	62	4	M	Left	PIGD	24-10		x	x
12	57	11	M	Right	TD	28-13		x	x
13	59	9	M	Left	TD	41-21		x	x

1. Thenganatt, M. A. & Jankovic, J. Parkinson disease subtypes. *JAMA Neurol* 71, 499–504 (2014).

* denotes three patients (pts 8-10) where the 20 Hz stimulation was not delivered due to time limitations.

Supplementary section: Simulations with 2nd order LTI system (damped oscillator)

We have implemented an LTI system with an impulse response similar to the ECA waveform (damped oscillation) and a spectral content peaking at ~300Hz. We convolved 5-second-long impulse trains with 20, 130, 160 and 180Hz through this system and observed the output (Figure S6). As illustrated, there was no bandlimited HFO induced in any condition. However, there is a power increase around the harmonics of the pulse frequency as expected (i.e., since ECA is time locked to the stimulation pulses). The simulated spectrum resembled the in vivo neural recordings (Figure S7) only when an additive bandlimited activity mimicking HFO was added to the simulation (Figure S8).

We further expanded this simulation and tested more resonant systems with different natural frequencies (270Hz, 310Hz and 350Hz). With the same setup, we convolved 5-second-long impulse trains with 130, 160 and 180Hz through these systems and observed the output. As we discussed in the manuscript in Figure 7, depending on at what phase we hit the resonant waveform (i.e., the impulse response of the underlying system), different frequencies induced more resonance. By increasing the resonance and natural frequency of the damped oscillator, ECA waveforms had changes in their phase and amplitude within the first few pulses. In fact, by adjusting the center frequency of the oscillator and thus the ECA phase where the next pulse hits, the behavior of the simulated ECA waveform could be manipulated (Figure S9 shows ECA amplification at 130 Hz, Figure S10 at 160 Hz and Figure S11 at 180 Hz), as we have observed in our LFP recordings. These simulations also provide a strong evidence for amplitude and phase adaptation over several consecutive stim pulses (Figure 6, Figure S7). The lack of adaptation in phase or amplitude with 20 Hz stimulation was the basis of our choice for using the phase of ECA after 20Hz stimulation in Figure 7. The consecutive pulses are far away from each other and the signal dampens by the arrival of the next pulse. For these reasons, we argue that the non-resonant 20Hz stimulation could be used to infer the undistorted phase space of the ECA (which appears as a dampening oscillation) since there is enough delay between each consecutive pulse.

Overall, we observed that the 2nd order LTI system can reproduce the amplitude and phase adaptations in the ECA and the resulting spectra has large peaks around the stimulation frequency and its harmonics. It is fair to note that the damped oscillator model here is an oversimplification of the underlying circuitry and it is likely that there are multiple nested oscillators interacting with each other to produce the ECA response we observe in the brain. More complex models are needed to fully simulate the in vivo ECA behavior (e.g., resonance, adaptation, delay…) and are out of scope of this study. A script to generate the simulations provided in this section is provided as a supplementary code file.

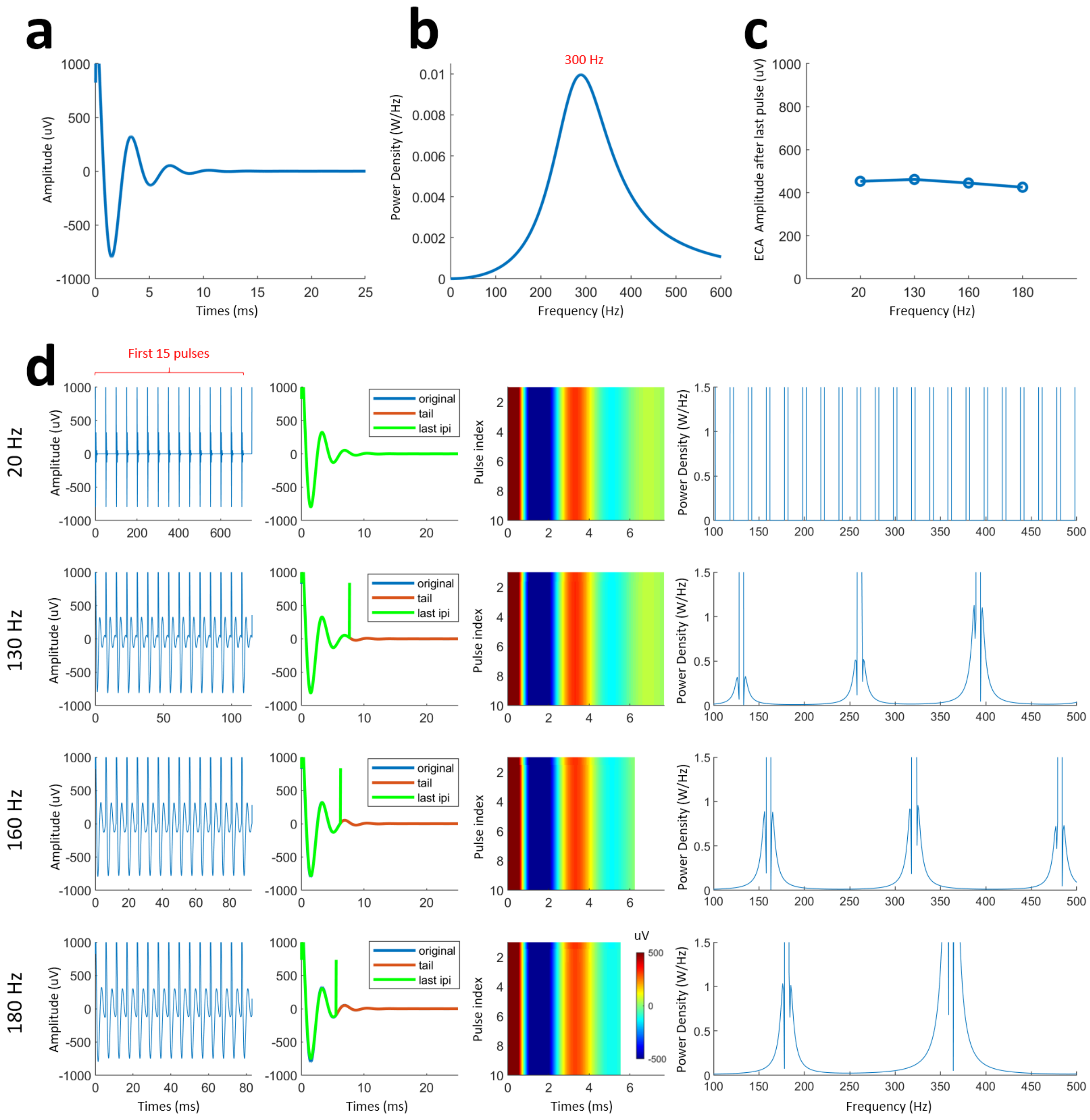

Figure S6: Simulation of a realistic 20Hz ECA. An LTI system with impulse response (a) similar to the ECA waveform observed after 20 Hz stimulation and a spectral content (b) peaking at ~300Hz was implemented. Five-second-long impulse trains at 20, 130, 160 and 180 Hz were passed through the system for comparison. (c) The amplitude of the first peak of ECA waveform after the last pulse was similar between different frequencies. (d) Details of the output of the LTI system with different frequencies (rows labeled accordingly). The first column shows the first 15 pulses and illustrates the amplitude change after each pulse. The second column compares the original impulse response and the response of the final pulse. The last inter-pulse interval (ipi) is also presented to show at what phase the pulse hits the original waveform. The third column illustrates the ECA waveform after the first 10 pulses, to investigate adaptation in delay of the first ECA peak. Color limits are identical to the bottom one. The first three columns illustrate that the ECA response was similar in amplitude and delay when the frequency of pulses was changed. The last column demonstrates the spectral content of the output, which was devoid of any bandlimited HFO activity. The only increase in spectral power in this band was localized around the harmonics of the stimulation artifact as expected (i.e., since ECA is time locked to the stimulation pulses).

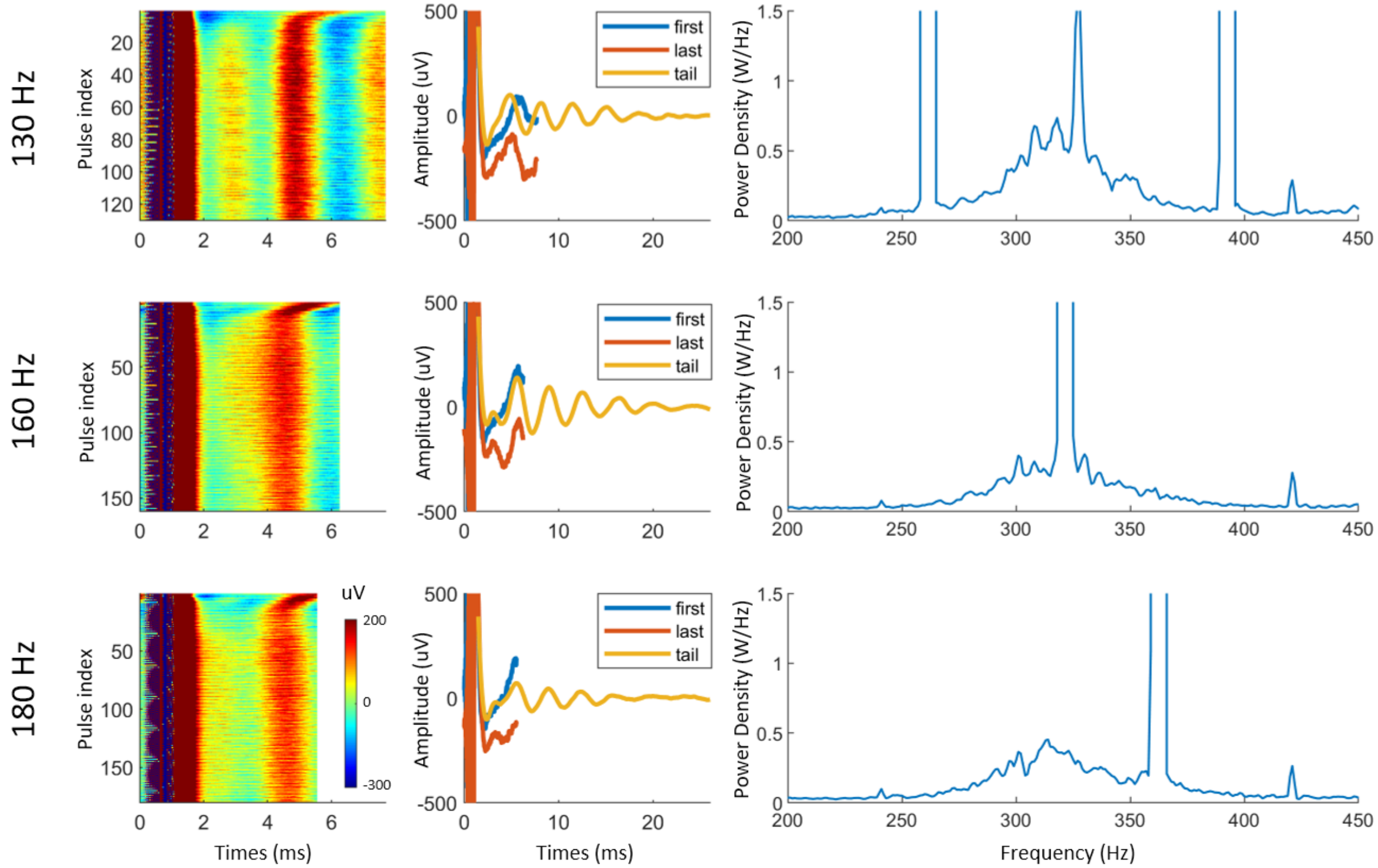

Figure S7: The ECA waveform and HFO response to different high-frequency stimulations (rows labeled) in a representative patient. The first column shows the first 150 pulses and illustrates the amplitude change after each pulse. The second column shows the adaptation over the whole 22s stimulation, as the settling took longer. The third column compares the response of the first and last inter-pulse intervals as well as the final pulse. Color limits are identical to the bottom one. The first three columns illustrate that the ECA adapts in amplitude and delay over different temporal scales when the frequency of the stimulation was changed. It is likely that there are multiple nested oscillators interacting with each other to produce the ECA response observed in the brain. The last column demonstrates the spectral content with bandlimited HFO activity, which is present in all three instances, regardless of the stimulation frequency.

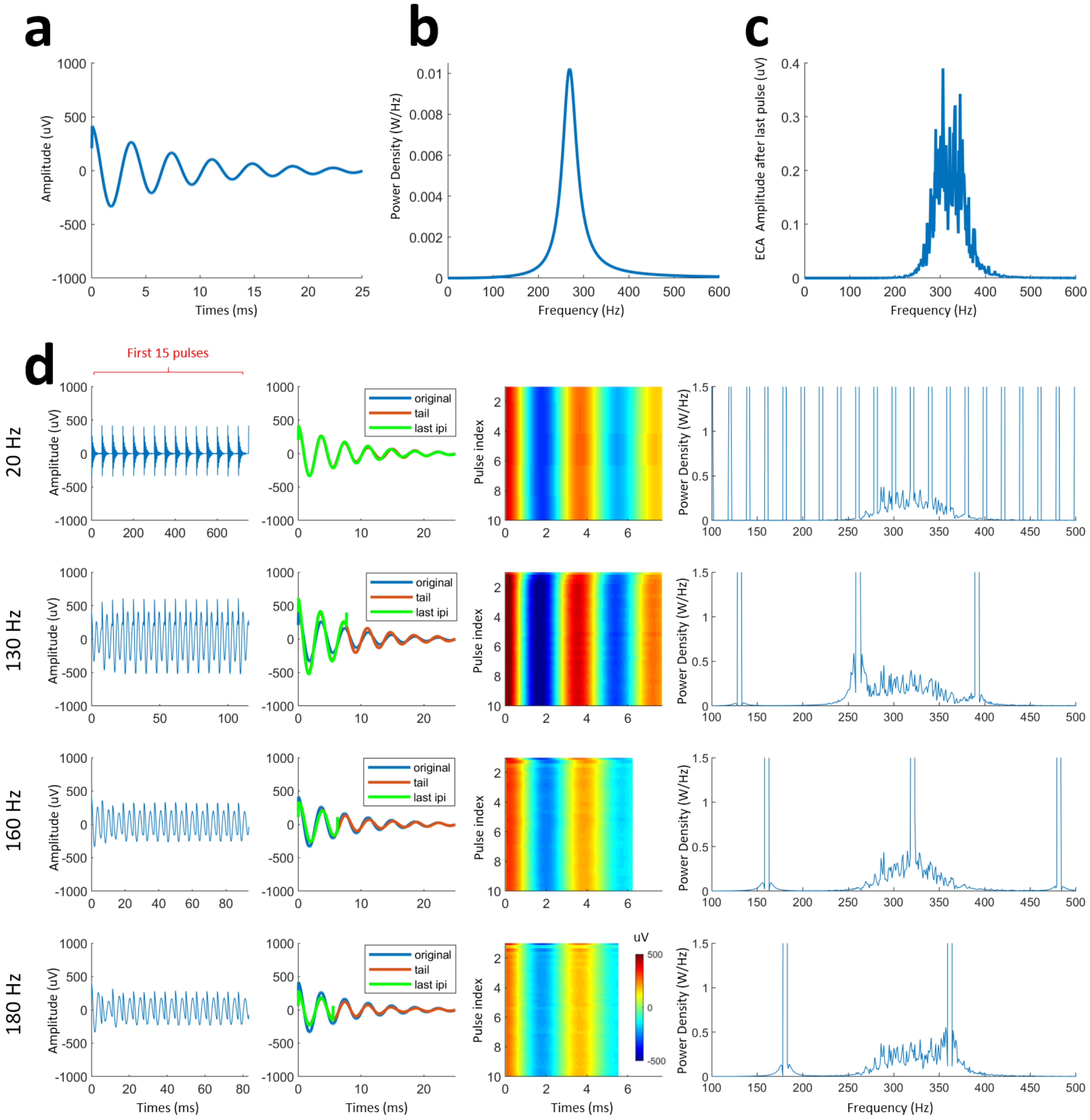

Figure S8: Simulation of a ECA waveform that with higher resonance and added independent bandlimited noise. Similar to Figure S6, the pulse trains at different frequencies are passed through an LTI system with impulse response (a) and a spectral content (b) peaking at ~270Hz. (c) Bandlimited white noise similar to the HFO activity seen in neural data is added to the output of the LTI system. (d) This system amplified the ECA waveform at 130 Hz but damped it at 160 and 180 Hz. The amplitude changes can be seen in the first and second columns. Additionally, the adaptation in delay of the first ECA peak is observed in the third column. Color limits are identical to the bottom one. While no adaptation is observed at 20 Hz, it takes 2-3 pulses for the ECA waveform to adapt to its final form. The power spectral density plots on the fourth column resemble the bandlimited HFO activity seen in the neural data.

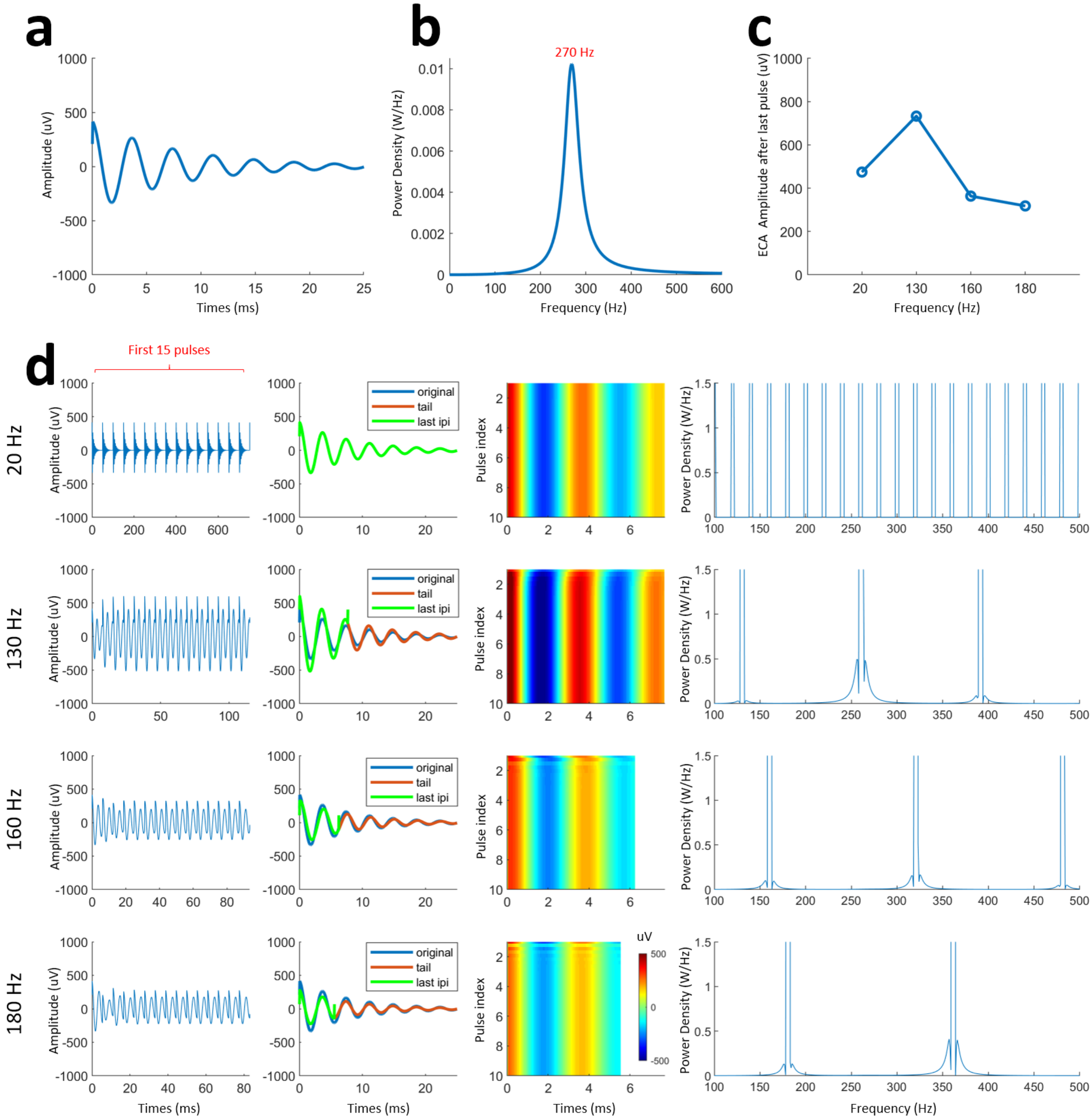

Figure S9: Simulation of a ECA waveform that with higher resonance and center frequency of 270Hz. Similar to Figure S6, the pulse trains at different frequencies are passed through an LTI system with impulse response (a) and a spectral content (b) peaking at ~270Hz. (c) This system amplified the ECA waveform at 130 Hz but damped it at 160 and 180 Hz. (d) The amplitude changes can be seen in the first and second columns. Additionally, the adaptation in delay of the first ECA peak is observed in the third column. Color limits are identical to the bottom one. While no adaptation is observed at 20 Hz, it takes 2-3 pulses for the ECA waveform to adapt to its final form. The power spectral density plots on the fourth column still lack the bandlimited HFO activity seen in the neural data, compared to Figure S8 with added bandlimited noise.

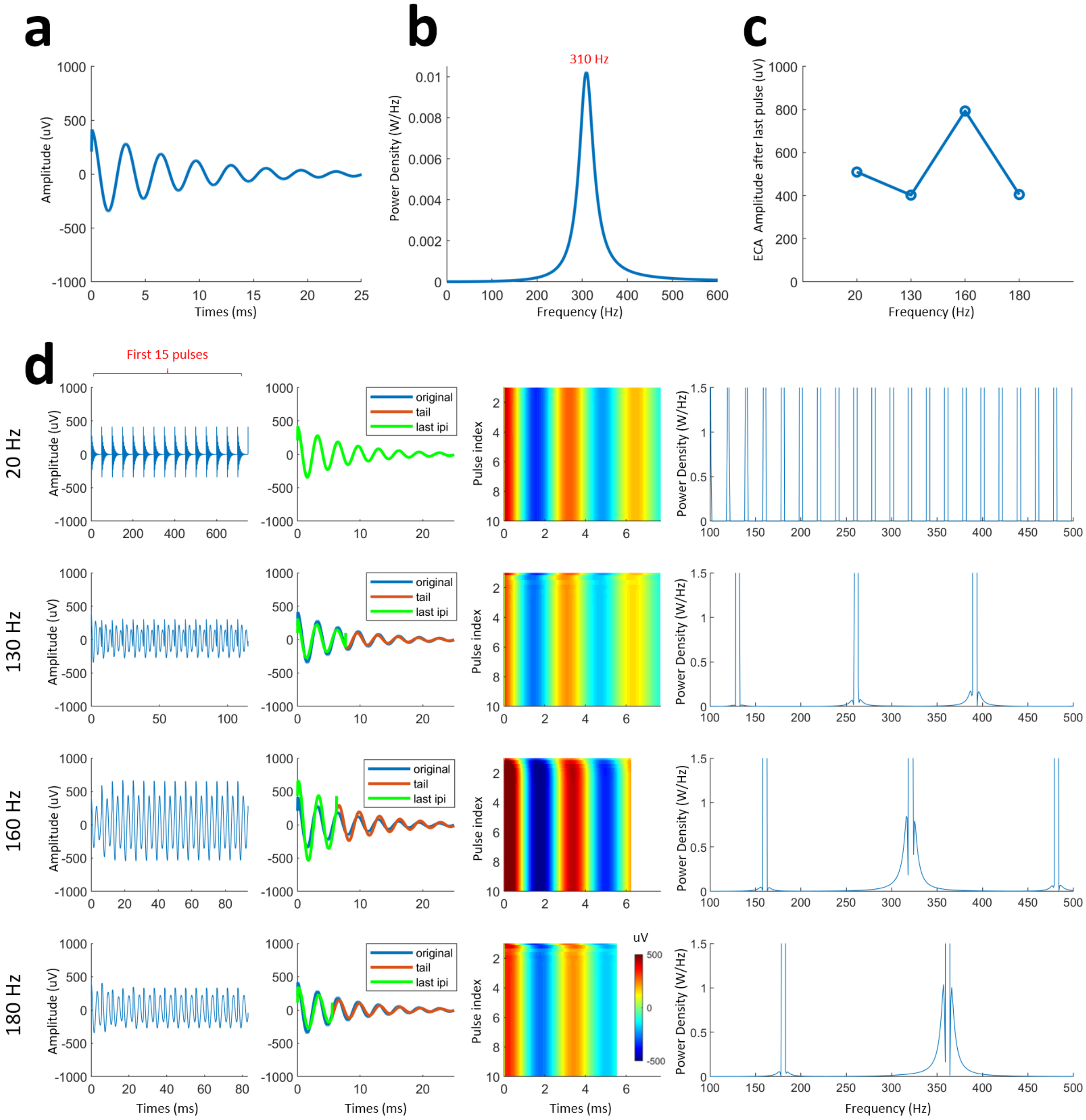

Figure S10: Simulation of a ECA waveform that with higher resonance and center frequency of 310Hz. Similar to Figure S6, the pulse trains at different frequencies are passed through an LTI system with impulse response (a) and a spectral content (b) peaking at ~310Hz. (c) This system amplified the ECA waveform at 160 Hz but damped it at 130 and 180 Hz. (d) The amplitude changes can be seen in the first and second columns. Additionally, the adaptation in delay of the first ECA peak is observed in the third column. Color limits are identical to the bottom one. While no adaptation is observed at 20 Hz, it takes 2-3 pulses for the ECA waveform to adapt to its final form. The power spectral density plots on the fourth column still lack the bandlimited HFO activity seen in the neural data.

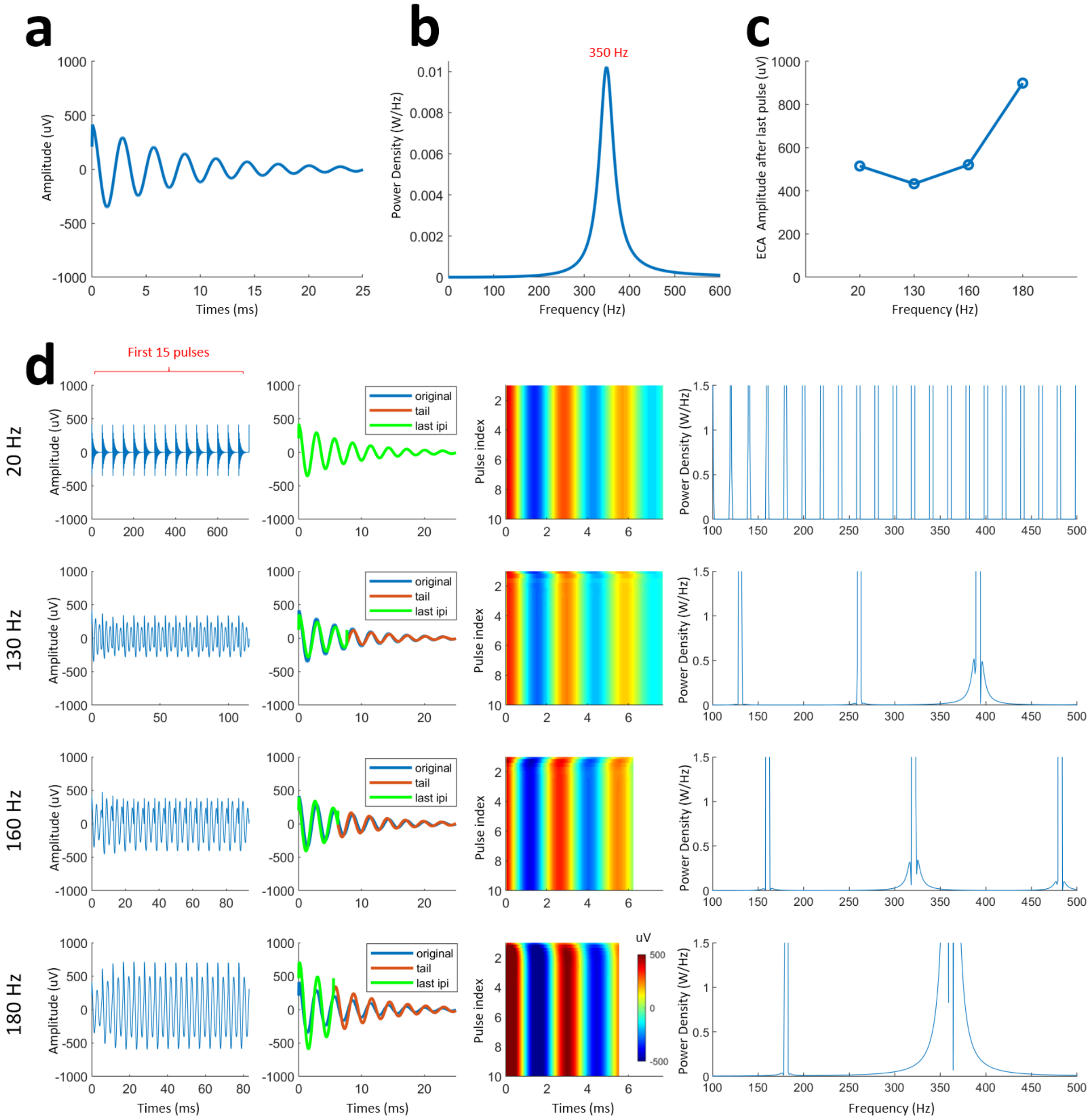

Figure S11: Simulation of a ECA waveform that with higher resonance and center frequency of 350Hz. Similar to Figure S6, the pulse trains at different frequencies are passed through an LTI system with impulse response (a) and a spectral content (b) peaking at ~350Hz. (c) This system amplified the ECA waveform at 180 Hz but damped it at 160 and 130 Hz. (d) The amplitude changes can be seen in the first and second columns. Additionally, the adaptation in delay of the first ECA peak is observed in the third column. Color limits are identical to the bottom one. While no adaptation is observed at 20 Hz, it takes 2-3 pulses for the ECA waveform to adapt to its final form. The power spectral density plots on the fourth column still lack the bandlimited HFO activity seen in the neural data.
