# Supplementary material for: Electroceutically induced subthalamic high-frequency oscillations and evoked compound activity may explain the mechanism of therapeutic stimulation in Parkinson’s disease
Source: Commun Biol. 2021 Mar 23;4:393. doi: 10.1038/s42003-021-01915-7 (PMC7988171; doi:10.1038/s42003-021-01915-7)
Supplement: Supplementary file 2 — Description of Additional Supplementary Files [file 42003_2021_1915_MOESM2_ESM.pdf]

Description of Additional Supplementary Files

File name: Supplementary Data 1

Description: Raw data points for the figures 2c, d, g; 3b, d; 4e,; 5c, d, e; 6b; S1.

File name: Supplementary Code

Description: The Matlab code for the ECA simulations used to generate the figures available in the supplementary section: “Simulations with 2nd order LTI system - damped oscillator.
