# Supplementary material for: Electroceutically induced subthalamic high-frequency oscillations and evoked compound activity may explain the mechanism of therapeutic stimulation in Parkinson’s disease
Source: Commun Biol. 2021 Mar 23;4:393. doi: 10.1038/s42003-021-01915-7 (PMC7988171; doi:10.1038/s42003-021-01915-7)
Supplement: Supplementary file 4 — Supplementary Code [file 42003_2021_1915_MOESM4_ESM.pdf]

Supplementary Code: *The script to reproduce the simulation of damped oscillator experiments. Please save the contents as an .m file to run in Matlab. User adjustable parameters are given in the first cell.*

```
%% Sample script to generate simulation of ECA and HFO response
% Part of supplementary Information for “Ozturk et al., Electroceutically induced
% subthalamic high-frequency oscillations and evoked compound activity may
% explain the mechanism of therapeutic stimulation in Parkinson’s disease”
%
%
% User parameters
add_noise = 0; % 0 or 1, whether to add bandlimited noise

% filt_lim affects resonance, narrow bw will increase resonance
% uncomment below for the figures in the supplementary material
filt_lim=[220 380]; % realistic ECA
% filt_lim=[250 290]; % 130 favoring
% filt_lim=[290 330]; % 160 favoring
% filt_lim=[330 370]; % 180 favoring

%% No need to change below this point
Fs=30000; % sampling frequency
wd=Fs; ov=round(wd*0.5); % window and overlap for PSD estimation

% generate a 5 second long signal with one pulse
delta=zeros(5*Fs,1);
delta_gain=5e4; % multiply to produce ECA at similar amplitudes to neural data in uV, so
similar psd scales
delta(1)=delta_gain;

% generate the additive noise
noise_gain=50*add_noise;
nn=wgn(length(delta),1,1);
[b1,a1]=butter(2,[280 360]/(Fs/2));
noise_add=noise_gain*filter(b1,a1,nn);
[Pn,Fn]=pwelch(noise_add,wd,ov,wd,Fs); % spectral analysis

% LTI system to model a damped oscillation
[b,a]=butter(1,filt_lim/(Fs/2));
ecap=filter(b,a,delta);
[Pw,Fw]=pwelch(ecap,wd,ov,wd,Fs); % spectral analysis

ff=figure('pos',[0 900 1200 250]);
subplot(1,3,1)
plot((1:length(ecap))/Fs*1e3,ecap,'linew',2);  box off
xlim([0 25]); ylim([-1000 1000])
% xlabel('Times (ms)'); ylabel('Amplitude (uV)')

subplot(1,3,2);
plot(f2,abs(Pw),'linew',2); box off
xlim([0 600]); ylim([0 0.0105])
% xlabel('Frequency (Hz)'); ylabel('Power Density (W/Hz)')

% feed the pulse trains
figure('pos',[0 0 1200 800])
freqs =[20 130 160 180]; % frequencies to test
num_freqs=length(freqs);
eca_amps=nan(1,num_freqs); % note the eca amplitude (1st peak) across frequencies

for k=1:num_freqs
    % generate the pulse train
    tr=delta;
    SFs=freqs(k);
```

```
    T=(1/SFs)*Fs;
    T=fix(T);
    tr(1:T:end)=delta_gain;
    ps=.030*Fs;
    tr(end-ps:end)=0; % leave the last 30ms for analysis of ECA after stimulation
    ix=find(tr, 1, 'last');
    pulse_num = floor(ix/T);

    eca=filter(b,a,tr)+noise_add; % feed the train and add noise

    eca_aligned=reshape(eca(1:pulse_num*T),T,pulse_num); % get inter-pulse segmented data

    [Pr,Fr]=pwelch(eca(1:end-ps),wd,ov,wd,Fs);  % spectral analysis - remove after ps,
since it was changed to 0

    % plot first 15 pulses
    subplot(num_freqs,5,5*(k-1)+1);
    plot((1:length(eca))/Fs*1e3,eca);xlim([0 T*15]/Fs*1e3)
    ylim([-1000 1000]); box off
    ylabel(num2str(freqs(k)),'fontsize',13)

    % plot the original and last eca, and last inter-pulse interval
    subplot(num_freqs,5,5*(k-1)+2);
    plot((1:length(ecap))/Fs*1e3,ecap); hold on;
    plot((1:length(eca(ix:end)))/Fs*1e3,eca(ix:end));
    plot((1:length(eca(ix-T:ix)))/Fs*1e3,eca(ix-T:ix))
    xlim([0 25]); ylim([-500 500]*2); legend('original','tail','last
ipi','location','ne'); box off
    eca_amps(k)=max(eca(ix+0.003*Fs:end))-min(eca(ix+0.003*Fs:end)); % first 3ms have the
artifact

    % plot the first 10 pulses in 2D for observing phase adaptation
    subplot(num_freqs,5,5*(k-1)+3);
    imagesc((1:length(eca_aligned(:,1)))/Fs*1e3,1:eca_aligned(1,:),eca_aligned');
colormap jet;
    ylim([1 10]); xlim([0 ceil(Fs/130)]/Fs*1e3); box off
    caxis([-5e2 5e2]);

    % plot the spectral content
    subplot(num_freqs,5,5*(k-1)+(4:5));
    plot(f2,Pr); box off
    ylim([0 3/2]); xlim([100 500]);
end
suptitle(num2str(filt_lim))

figure(ff)
if add_noise
    subplot(1,3,3);
    plot(f2,abs(Pn),'linew',2); box off
    xlim([0 600]);
else
    subplot(1,3,3);
    plot(eca_amps,'o-','linew',2); box off
    set(gca,'xtick',1:num_freqs,'xticklabel',freqs)
    xlim([0 num_freqs+1]); ylim([0 1000])
    %xlabel('Frequency (Hz)'); ylabel('Amplitude (uV)')
    suptitle(num2str(filt_lim))
end
```
