# Supplementary material for: Electroceutically induced subthalamic high-frequency oscillations and evoked compound activity may explain the mechanism of therapeutic stimulation in Parkinson’s disease
Source: Commun Biol. 2021 Mar 23;4:393. doi: 10.1038/s42003-021-01915-7 (PMC7988171; doi:10.1038/s42003-021-01915-7)
Supplement: Supplementary file 5 — Reporting Summary [file 42003_2021_1915_MOESM5_ESM.pdf]

nature research

Corresponding author(s): Nuri F. Ince

Last updated by author(s): 2/19/2021

Reporting Summary

Nature Research wishes to improve the reproducibility of the work that we publish. This form provides structure for consistency and transparency in reporting. For further information on Nature Research policies, see our Editorial Policies and the Editorial Policy Checklist.

Statistics

For all statistical analyses, confirm that the following items are present in the figure legend, table legend, main text, or Methods section.

n/a	Confirmed	
[ ]	[x]	The exact sample size (*n*) for each experimental group/condition, given as a discrete number and unit of measurement
[ ]	[x]	A statement on whether measurements were taken from distinct samples or whether the same sample was measured repeatedly
[ ]	[x]	The statistical test(s) used AND whether they are one- or two-sided<br>*Only common tests should be described solely by name; describe more complex techniques in the Methods section.*
[x]	[ ]	A description of all covariates tested
[ ]	[x]	A description of any assumptions or corrections, such as tests of normality and adjustment for multiple comparisons
[ ]	[x]	A full description of the statistical parameters including central tendency (e.g. means) or other basic estimates (e.g. regression coefficient) AND variation (e.g. standard deviation) or associated estimates of uncertainty (e.g. confidence intervals)
[ ]	[x]	For null hypothesis testing, the test statistic (e.g. *F*, *t*, *r*) with confidence intervals, effect sizes, degrees of freedom and *P* value noted<br>*Give P values as exact values whenever suitable.*
[x]	[ ]	For Bayesian analysis, information on the choice of priors and Markov chain Monte Carlo settings
[x]	[ ]	For hierarchical and complex designs, identification of the appropriate level for tests and full reporting of outcomes
[x]	[ ]	Estimates of effect sizes (e.g. Cohen's *d*, Pearson's *r*), indicating how they were calculated

Our web collection on statistics for biologists contains articles on many of the points above.

Software and code

Policy information about availability of computer code

Data collection	Custom Simulink models were developed in Matlab 2014a for the data collection. The drivers and API (application programming interface) of gHiAmp (gTec, Austria) and Grapevine (Ripple, UT, USA) were used for communication and control of these commercially available devices with a laptop running Windows 7. The versions of the commercial software used are listed below:<br>1- g.HIsys Highspeed Online Processing for SIMULINK ver. 3.15.02_Win64<br>2- g.HIamp Driver ver. 2.16.01_Win64<br>3- NIP processor firmware ver. 1.8.2<br>4- Trellis ver. 1.8.2.291-x86
Data analysis	Matlab 2018a was used for both signal processing and statistical analyses. All scripts were developed in-house, except Circular Statistics Toolbox by P. Berens 2009 (cited in the manuscript). The key Matlab functions utilized are noted with the corresponding input parameters.

For manuscripts utilizing custom algorithms or software that are central to the research but not yet described in published literature, software must be made available to editors and reviewers. We strongly encourage code deposition in a community repository (e.g. GitHub). See the Nature Research guidelines for submitting code & software for further information.

Data

Policy information about availability of data

All manuscripts must include a data availability statement. This statement should provide the following information, where applicable:

- Accession codes, unique identifiers, or web links for publicly available datasets
- A list of figures that have associated raw data
- A description of any restrictions on data availability

The data that support the findings of this study are available on request from the corresponding author. The raw data are not publicly available as the data might

contain potentially identifying or sensitive information that could compromise the privacy of the research participants.
The code for the ECA simulations is provided as a supplementary material.
The data for the figures 2c, d, g; 3b, d; 4e,; 5c, d, e; 6b; S1 is provided in the supplementary data as an MS Excel table.

Field-specific reporting

Please select the one below that is the best fit for your research. If you are not sure, read the appropriate sections before making your selection.

- [x] Life sciences
- [ ] Behavioural & social sciences
- [ ] Ecological, evolutionary & environmental sciences

For a reference copy of the document with all sections, see nature.com/documents/nr-reporting-summary-flat.pdf

Life sciences study design

All studies must disclose on these points even when the disclosure is negative.

Sample size	16 independent hemispheres. The patient recruitment was stopped when a particular experiment achieved significance.
Data exclusions	No data was excluded from the analyses. Number of subjects performing a particular experiment is clearly stated where applicable.
Replication	The same experimental paradigm was performed on multiple subjects to verify the neuromodulatory effect of the high- and low-frequency stimulation. Additionally, the same stimulation was delivered in and out of target structure over multiple subjects to verify that the observed effects are not due to hardware or stimulation artifacts.
Randomization	Randomization was not relevant to our study as we did not contrast any of the experimental parameters between groups.
Blinding	Blinding was not relevant to our study since the experimental parameters were not chosen/optimized per subject.

Reporting for specific materials, systems and methods

We require information from authors about some types of materials, experimental systems and methods used in many studies. Here, indicate whether each material, system or method listed is relevant to your study. If you are not sure if a list item applies to your research, read the appropriate section before selecting a response.

Materials & experimental systems

n/a	Involved in the study	
x		Antibodies
x		Eukaryotic cell lines
x		Palaeontology and archaeology
x		Animals and other organisms
	x	Human research participants
x		Clinical data
x		Dual use research of concern

Methods

n/a	Involved in the study	
x		ChIP-seq
x		Flow cytometry
x		MRI-based neuroimaging

Human research participants

Policy information about studies involving human research participants

Population characteristics	Thirteen patients (two females) with PD undergoing awake bilateral STN-DBS implantation were included in the study. Their ages ranged from 53 to 70 (mean ± standard deviation = 60.5±4.7). Recordings from three patients were obtained bilaterally and the rest of the patients were recorded unilaterally from the hemisphere contralateral to the most affected side.
Recruitment	Patients with PD undergoing awake bilateral STN-DBS implantation at St. Luke's Medical Center of Baylor College of Medicine were included in the study. There were no other criteria for patient recruitment.
Ethics oversight	The study protocol was approved by the Institutional Review Boards of Baylor College of Medicine and University of Houston. This information is provided in the section "Methods", subsection "Patients".

Note that full information on the approval of the study protocol must also be provided in the manuscript.
